# Isoflavones impair anti-PD1 efficacy in breast cancer, regardless of dietary fiber or fecal short-chain fatty acid levels

**DOI:** 10.3389/fimmu.2026.1835466

**Published:** 2026-07-06

**Authors:** Fabia de Oliveira Andrade, Kerrie B. Bouker, Melike Ozgul-Onal, Lu Jin, Idalia Cruz, William Helferich, Audrey Gao, Karla Andrade de Oliveira, Vivek Verma, Christopher Staley, Patricia L. Foley, Leena Hilakivi-Clarke

**Affiliations:** 1 The Hormel Institute, University of Minnesota, Austin, MN, United States; 2 Georgetown University Medical Center, Washington, DC, United States; 3 Department of Histology-Embryology, Medicine, Mugla Sitki Kocman University, Mentese, Mugla, Türkiye; 4 Department of Food Science and Human Nutrition, University of Illinois Urbana-Champaign, Urbana, IL, United States; 5 Department of Biochemistry and Pharmacology, Federal University of Piaui, Teresina, PI, Brazil; 6 Department of Surgery, Medical School, University of Minnesota, Minneapolis, MN, United States; 7 Depatment of Food Science and Nutrition, University of Minnesota, Minneapolis, MN, United States

**Keywords:** anti-PD1, genistein, gut microbiome, microbiota assessable carbohydrates (MACs), tamoxifen

## Abstract

**Background:**

Fermentable dietary fibers, or microbiota-accessible carbohydrates (MACs), are hypothesized to enhance responsiveness to immune checkpoint blockade (ICB) therapy in breast cancer (BC) by increasing fecal short-chain fatty acid (SCFA) production. However, existing research findings have been inconsistent. Given that hormone-sensitive breast cancer is highly influenced by estrogen levels, the presence of estrogenic isoflavones in certain MAC sources may partially account for these discrepancies. Consequently, investigating the roles of isoflavones versus MACs in BC models is warranted.

**Methods:**

C57BL/6Tac mice were fed low-MAC (AIN93G), low-MAC supplemented with isoflavone genistein, high-MAC (5V5M), or high-MAC isoflavone (high-MACi; 5058D) diet to assess anti-PD1 efficacy against E0771 triple-negative breast cancer (TNBC) and 7,12-dimethylbenz[a]anthracene (DMBA)-initiated estrogen receptor α-positive (ERα^+^) mammary tumors. Effects of blocking ERα with tamoxifen (TAM) and dietary impact on the gut microbiome and immune signaling (NanoString) were also evaluated.

**Results:**

High-MAC diets increased fecal microbial diversity, the abundances of SCFA-producing families, and fecal SCFA levels, compared with the low-MAC diet. Anti-PD1 was effective in TNBC models with high-MAC or low-MAC diets, but responsiveness was eliminated by the inclusion of isoflavones (high MACi) or genistein (low MAC). Anti-PD1 reduced exhausted CD8^+^ T cells in high-MAC-fed mice but increased them in high-MACi-fed mice. ERα^+^ tumors were resistant to anti-PD1. TAM induced sensitivity to anti-PD1 in both TNBC and ERα^+^ models possibly by modulating TH17 pathways.

**Conclusions:**

Our results highlight the role of diet in impacting the effectiveness of ICB therapies. Increased SCFA alone is not predictive of response to anti-PD1, but if the tumor expresses ERα or if the diet contains ERα-activating compounds, such as isoflavones, blocking ERα^+^ might convert unresponsive tumors responsive to anti-PD1.

## Introduction

Factors determining the effectiveness of cancer immunotherapies, such as those targeting programmed cell death protein 1 (PD1) and its ligand PDL1, remain elusive. Diet might influence responsiveness: observational evidence, confirmed in animal studies, suggests that melanoma patients consuming high levels of dietary fiber are most responsive to immunotherapies while prebiotics may have an opposite effect (1). Beneficial effects of fiber on immune responses, including PD1/PDL1 therapies, are thought to be mediated by a fiber-induced increase in the abundance of gut bacteria that produce short-chain fatty acids (SCFAs) (2–4). However, the beneficial role of dietary fiber in anti-PD1 or anti-PDL1 therapy has been recently challenged by a study involving diverse murine tumor models (5). Breast cancer was not among the models.

Microbiota-accessible carbohydrates (MACs) are a group of fermentable dietary fibers (6) that increase gut bacterial production of SCFAs (7). However, many high-MAC diets that are fed to laboratory mice contain the estrogenic isoflavones genistein and daidzein, mainly in glycoside form (8). When high-MACi diets are fed to laboratory mice, elevated serum and urine levels of genistein and daidzein as well as equol (metabolite of daidzein) have been reported, compared with diets containing only trace amounts of isoflavones, such as the low-MAC AIN93G diet (8). Isoflavones in the diet might modify immune therapy response (9). Human dietary sources of MACs, such as vegetables, legumes, fruits, and berries, also contain high levels of isoflavones. It has been reported that activated estrogen receptor α (ERα) promotes immunosuppression (10, 11). Estrogens also drive gender-dependent immunosuppression in the tumor microenvironment (TME) (12), including driving differentiation of naive CD4^+^ T cells into immunosuppressive Treg cells (13): these effects are purported to explain why men might respond better to cancer immunotherapies than women (14). Furthermore, inhibiting ERα with fulvestrant improved response to immunotherapies against estrogen-insensitive, preclinical, non-breast tumor models (10, 15).

We investigated here whether feeding mice high- or low-MAC diets, or a high-MAC diet containing isoflavones from soymeal (high-MACi), affected responsiveness to anti-PD1 therapy against triple-negative breast cancer (TNBC) or ERα^+^ mammary tumors in female mice. In addition, the impact of blocking ERα^+^ with tamoxifen (TAM) on ICB effectiveness was studied. Our results indicated that high-MAC and high-MACi diets increased fecal SCFA-producing bacteria and SCFA levels. However, high fecal SCFA levels alone did not predict responsiveness to anti-PD1, since anti-PD1 reduced TNBC growth in mice fed high- or low-MAC diet but not in mice fed a high-MACi diet. The latter observation suggests that isoflavones impaired ICB’s effect even in mice that had high fecal SCFA levels. TAM converted TNBCs in high-MACi-fed mice towards responsiveness, while ERα^+^ mammary tumors started responding to anti-PD1 if mice were fed a low-MAC diet and treated with TAM. Thus, TAM’s effect was dependent on tumor hormone receptor status and the MAC diet mice were consuming.

## Materials and methods

### Mice

Four-week-old, female C57BL/6 mice were obtained from Taconic Biosciences, Inc. The studies were performed at Georgetown University and at the University of Minnesota, keeping mice at 22 °C ± 2°C with *ad libitum* access to diet and chlorinated filtered drinking water, and approved by their respective Institutional Animal Care and Use Committees. At Georgetown University, mice were maintained on a 12-h light–dark cycle in a specific pathogen-free facility on HEPA-filtered ventilated racks, while at the University of Minnesota, mice were housed in a conventional facility in static cages with filter tops on a 14:10-h light/dark cycle. Mice were euthanized at the end of the study using carbon dioxide inhalation (70% flow rate of CO_2_ per chamber volume), and tissues were harvested as needed.

### Diets

For studies 1 and 2, mice were fed irradiated diets that were either low MAC with no isoflavones (LabDiet AIN93G, Land O’Lakes Inc., Arden Hills, MN, USA), high MAC containing low levels of isoflavones (LabDiet 5V5M), or high MAC containing moderate levels of isoflavones (LabDiet 5058D). For study 3, mice were fed a low-MAC diet (AIN93G; TD.97184) or a low-MAC diet supplemented with 500 ppm of genistein (AIN93G + 500 ppm of genistein; TD.210530) from Envigo Teklad Diets. According to Envigo Teklad Diets, the high-MAC isoflavone (high-MACi) diet contained 125–275 ppm of these isoflavones. Similar levels have been reported in a study that compared isoflavone levels in several different laboratory diets, including PMI 5058 and AIN93G diets (16). Basic nutritional comparisons of these diets are provided in **Supplementary Table 1**. While the diets were not nutritionally identical, the percentage and source of protein, fat, and carbohydrate in the three diets were roughly similar. Mice were randomized to dietary groups by body weight to ensure that initial weights were similar among all groups. Genistein was provided by Dr. William Helferich and had a purity of 99.09%, as measured by HPLC.

### Gut microbiome analysis: amplicon sequencing and bioinformatic processing

Fecal pellets were collected from mice fed low-MAC, high-MAC, or high-MACi diets for 4 weeks. These mice did not have any mammary tumors, and the mice used for this analysis are derived from study 2, which was performed at Hormel Institute-UMN. The V4 hypervariable region of the 16S rRNA gene was amplified and sequenced using the 515F/806R primer set by the University of Minnesota Genomics Center (UMGC) (17). Raw sequence data have been deposited in the NCBI SRA under BioProject accession number SRP579135. Sequence data were processed using mothur (ver. 1.41.1) (18) and a modified version of our previously published pipeline (19).

### Assessment of fecal SCFA levels

Fecal samples were collected and snap-frozen from mice fed low-MAC, high-MAC, or high-MACi diets from study 2. To obtain sufficient material, feces from two to three mice were pooled, yielding three biological replicates per group. All SCFA analytical standards were purchased from Sigma-Aldrich (USA). Fecal SCFA analysis was performed using a gas chromatography-coupled mass spectrometry (GC-MS) platform as previously described (20). Quantitation of SCFAs from the raw GC-MS data was performed using a 7-point calibration curve with appropriate solvent blanks, negative controls, and quality control samples run throughout the assessment and by utilizing the open-source software Skyline (21). Calibration curves were fitted independently for each compound by linear in log space regression using the peak ratio of each compound to the global internal standard. All calibration curves were fit with an *R*^2^ of at least 0.995 precision. All estimated sample concentrations were normalized by the starting lyophilized material weight.

### Effect of low- and high-MAC diets and tamoxifen on anti-PD1 response in the TNBC model

We investigated whether the three laboratory diets affected the growth of ERα-negative murine E0771 mammary tumors in syngeneic C57BL/6NTac mice. After acclimation (72 h), mice were assigned to low-MAC (*n* = 33), high-MAC (*n* = 33), or high-MACi (*n* = 32) diets. At 8 weeks of age, after 4 weeks on the diet, mice were allografted with 1 × 10^6^ E0771 cells in medium:Matrigel (1:1) into both fourth mammary fat pads. When tumors reached 50–70 mm³, mice were subdivided into four treatment groups (eight to nine mice): control IgG (Ctrl-IgG), tamoxifen (TAM) pellet (subcutaneous insertion; 5 mg, 60-day release, Innovative Research of America, USA), anti-PD1 mAb (125 µg IP; clone RMP1-14, BioXcell, USA), or TAM+anti-PD1. Anti-PD1 was administered in three doses at 3–4-day intervals; controls received a sham pellet and rat IgG (clone 2A3, BioXcell) on the same schedule. IgG and anti-PD1 antibodies were dissolved in the recommended buffers with pH 6.5 and pH 7.0, respectively (IP0070 and IP0065; BioXcell, USA). Mice that did not develop palpable tumors, or those with tumors that failed to reach the inclusion size (50–70 mm³), were excluded from the treatment cohort.

To account for enhanced immune activation from dual tumor allografting (22, 23), a second experiment evaluated diet effects in mice bearing a single tumor. Here, 0.5 × 10^6^ E0771 cells in 1:1 PBS: Matrigel (Corning, USA) were implanted into one mammary fat pad (9–10 mice/group). Upon reaching 50–70 mm³, mice received either three doses of 50 µg anti-PD1 or rat IgG control (Ctrl-IgG); no hormone therapy was used. Tumors in both studies were measured twice weekly, volumes calculated as ½ × (length × width²), and mice were monitored for up to 4 weeks after the final treatment or euthanized earlier if tumor burden exceeded 1,500 mm³.

### Effect of genistein on anti-PD1 response

C57BL/6NTac mice were assigned to either a low-MAC diet or the same diet supplemented with 500 ppm of genistein (genistein group; genistein was prepared in Dr. Helferich’s lab) for 4 weeks. Mice were allografted with 0.5 × 10^6^ E0771 cells in 1× PBS mixed with Matrigel (1:1) into the right fourth mammary fat pad. Once tumors reached 50–70 mm^3^, mice (*n* = 10/group) were treated with either control IgG (Ctrl-IgG) or anti-PD1 mAb. This experiment was repeated twice. In the first study, mice were treated with 150 µg of anti-PD1 mAb or Ctrl-IgG, and in the replicate, a reduced 100-µg anti-PD1 or Ctrl-IgG was used.

### Effects of low- and high-MAC diets on anti-PD1 therapy in the ERα^+^ mammary tumor model

This study evaluated whether DMBA-induced ERα^+^ mouse mammary tumors respond to anti-PD1 therapy and whether adding TAM modifies this response. Unlike rats, mice with ERα^+^ DMBA-induced tumors do not exhibit tumor growth reduction with TAM (24). Female C57BL/6NTac mice were fed low- or high-MAC diets from age 4 weeks and received medroxyprogesterone acetate (MPA, Mylan Pharmaceuticals, USA) at week 6, followed by 1 mg of DMBA (10 mg/mL dissolved in corn oil; Sigma-Aldrich, USA) doses at weeks 7–10, generating nearly 100% tumor incidence. Once tumors reached 50–70 mm³ (~12.6 weeks after the last DMBA dose), mice were divided into four treatment groups: 1) 200 μg of Ctrl-IgG and placebo pellet, 2) 200 μg of anti-PD1, 3) TAM (5 mg as a 60-day release pellet), or 4) a combination of anti-PD1 and TAM. Each group contained 7–11 mice. Higher anti-PD1 doses were used, anticipating a weak response in this ERα^+^ mammary tumor model. Tumor growth was monitored twice weekly over 11 weeks.

Anti-PD1 dosing strategies were tailored to match the specific baseline therapeutic sensitivities of the two tumor models. For the carcinogen-induced ER^+^ DMBA mammary tumor model, a standard dose regimen of 200 µg was selected due to its established resistance profile of ER^+^ breast cancers to single-agent checkpoint blockade (25, 26). For the syngeneic E0771 TNBC studies, empirical down-titration (150 µg down to 50 µg) was utilized across experiments given the model’s high sensitivity to anti-PD1 (27, 28). This approach avoided a therapeutic ceiling effect, ensuring an adequate experimental window to observe secondary combinatorial effects from dietary or hormonal interventions.

### Immune cell analysis in the E0771 mammary tumor model

**Figure 1:**
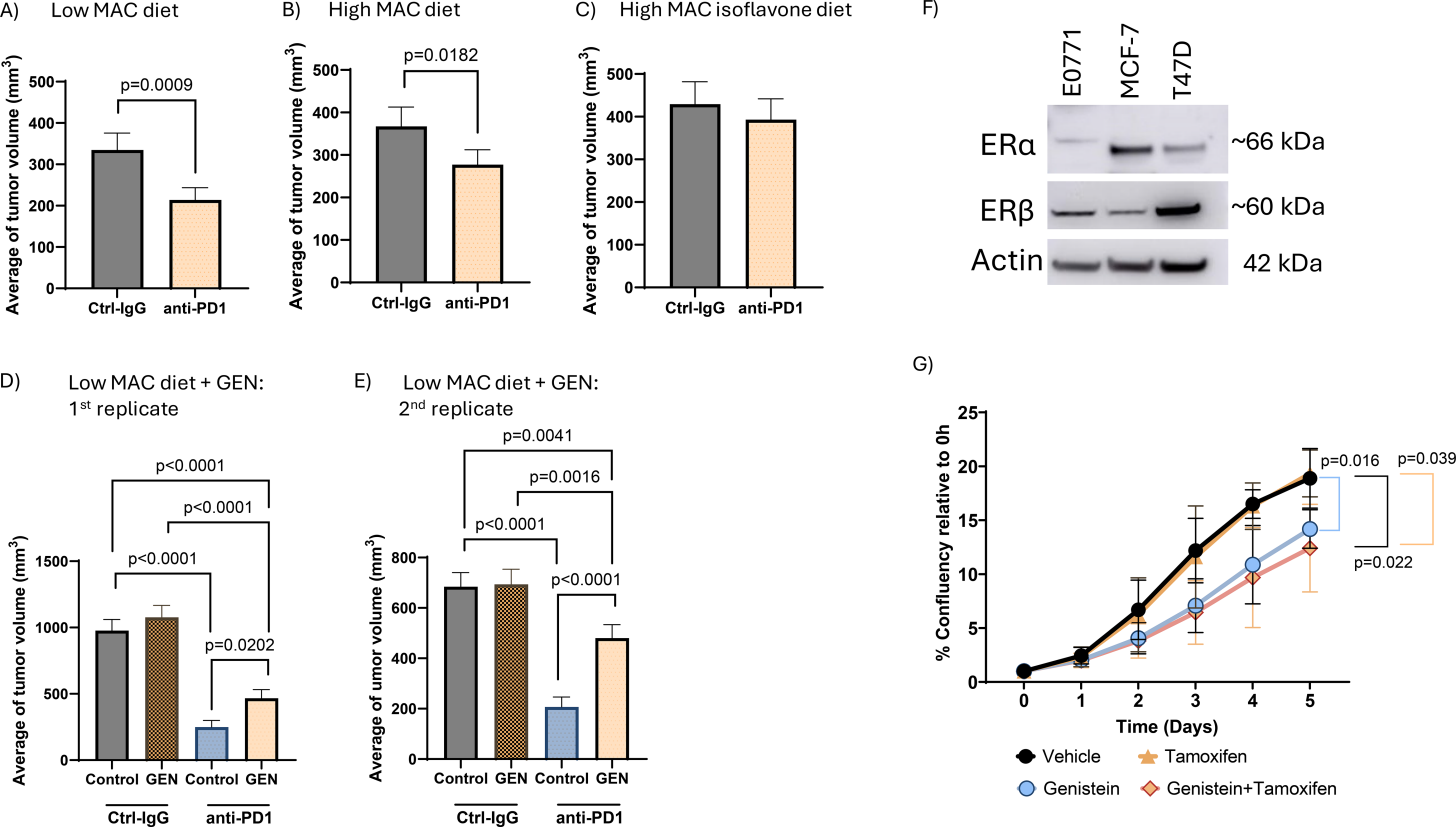
Effect of genistein (GEN) on anti-PD1 response in vivo, and the growth of E0771 TNBC cells in vitro. Mice were fed **(A)** low-MAC, **(B)** high-MAC, **(C)** high-MAC isoflavone (high-MACi) diet, or **(D, E)** low-MAC diet supplemented with 500 ppm of GEN. Each mouse was allografted with one tumor on the right fourth mammary gland. Tumor burden was calculated as an average of the measurement period per mouse, according to the R package agricolae. There were 7–10 mice in each group. Data are shown as mean ± SEM with statistical significance obtained by two-way repeated measures ANOVA followed by Student–Newman–Keuls post hoc test. **(F)** Protein levels of estrogen receptor α (ERα) and ERβ in E0771 murine mammary tumor cells and human ER+ MCF-7 and T47D breast cancer cell lines. **(G)** Growth of E0771 tumor cells treated with genistein (GEN: 5 μM), tamoxifen (TAM: 1 μM), a combination of genistein and tamoxifen, or vehicle control (0.04% DMSO) for up to 120 h. Data are shown as mean ± SEM of four independent experiments with three technical replicates each. Statistical significance shown was obtained by two-way repeated measures ANOVA followed by Student–Newman–Keuls post hoc test.

Tumors (*n* = 4–6/group) were harvested 12 days after the last anti-PD1 dose to assess the effect of diets on tumor-infiltrating immune cells. Tumor growth data in these mice are shown in **Figures 1A–C**. Single-cell suspensions were prepared by mechanical dissociation using 70-µm sterile nylon strainers and resuspended in T-cell media. Approximately 2 × 10^6^ cells were stimulated for 3 h at 37°C using 50 ng/mL of phorbol 12-myristate 13-acetate (Sigma-Aldrich, USA), 750 ng/mL of ionomycin (Sigma-Aldrich, USA), and 10 μg/mL of brefeldin (Biolegend, USA). Cells were stained for viability using the LIVE/DEAD Fixable Near IR Dead cell kit (Invitrogen, USA) and fixed with 4.2% formaldehyde buffer (BD Biosciences, USA). Immunophenotyping was performed using conjugated antibodies against CD3 (clone: 145-2C11; BD Biosciences, USA), CD8 (clone: 53-6.7; BD Biosciences, USA), TIM3 (clone: B8.2C12; Biolegend, USA), KLRG1 (clone: 2F1; Invitrogen, USA), and PD1 (clone: 29F-1A12; BD Biosciences, USA). Data were acquired using LSRFortessa Flow Cytometer and analyzed by FlowJo 10.9-10. Single cells were identified using FSC-H vs. FSC-A parameters. Within the singlet population, we sequentially gated for CD3^+^ and CD8^+^ T cells. Finally, the expression of IFNγ, TIM3, and PD1 was analyzed within the CD8^+^ subset. Positive staining gates were defined using Fluorescence Minus One (FMO) control, and compensation was performed using the AbC™ Total Antibody Compensation Bead Kit (Invitrogen).

### Expression of ERα and ERβ in breast cancer cells

Murine E0771 (CH3 BioSystems, USA) and human MCF-7 and T47D (donated by Dr. Robert Clarke, Hormel Institute, University of Minnesota) breast cancer cells were cultured in RPMI 1640 medium (Corning, USA) supplemented with 5% FBS (Peak Serum, USA). Once MCF-7 and T47D cells reached 70% confluence, the medium was replaced with phenol red-free RPMI 1640 supplemented with 5% charcoal-stripped FBS for 5 days. Following hormone deprivation, the cells were treated with 10 nM of 17β-estradiol (E2; Cayman Chemical, USA) for 24 h. E2 was diluted in ethanol, with the final ethanol concentration kept below 0.1%.

Protein extraction and Western blotting were conducted as previously described (29), with non-specific binding blocked using 4% bovine serum albumin (Fisher Bioreagents, USA) and primary antibodies against ERα (ab16460; Abcam, USA) and ERβ (NB120-3577; Novusbio, USA) used at a 1:1,000 dilution.

### Effect of isoflavone genistein on TNBC growth *in vitro*

E0771 cells (1 × 10³) were seeded in a 96-well plate and, after 24 h, treated with genistein (5 μM), tamoxifen (1 μM; Cayman Chemical, USA), a combination of genistein and tamoxifen, or vehicle control [0.04% dimethyl sulfoxide (DMSO); Sigma-Aldrich, USA]. Independent experiments (*n* = 4) were performed with three technical replicates. Cell growth was monitored using the Incucyte SX-5 system (Sartorius, Germany) for up to 120 h, with confluence analyzed as a percentage relative to the initial measurement at 0 h.

### NanoString analysis

The PanCancer Immune Profiling Panel containing 770 genes, including 40 PanCancer reference genes, purchased from NanoString Technologies, was used to assess possible differences in immune signaling pathways among mice fed high-MACi diet and treated with Ctrl-IgG alone, anti-PD1 alone, TAM alone, or the combination of both anti-PD1 and TAM. For this analysis, we used three tumors from three mice per group, harvested at the end of the tumor monitoring period.

### Gene expression by PCR

To address the inherent statistical limitations and potential risk of false negatives associated with the NanoString sample size, key differentially expressed genes identified in the pathway analysis were validated via quantitative real-time PCR (qPCR) in mammary gland tissues using an expanded cohort (*n* = 4–7 mice per group) (29). Primers used in qRT-PCR analysis were designed using the IDT tool (Integrated DNA Technologies, Coralville, IA, USA; the primer sequence can be found in **Supplementary Table 2**).

### Data analysis

#### Microbiome data analysis

Alpha diversity was calculated as the Shannon index using mothur. Beta diversity was calculated using the Bray–Curtis dissimilarity matrices and visualized via ordination using principal coordinate analysis (PCoA). Differences in alpha diversity indices and relative abundances of taxa were determined using the Kruskal–Wallis test in XLSTAT (ver. 2020.5.1; Addinsoft, New York). Differences in community composition were determined using analysis of similarity (ANOSIM) (30), and taxa that were significantly correlated with either PCoA axis by Spearman correlation, using the corr.axes function in mothur, were overlaid on the plot. ANOSIM was prioritized over PERMANOVA to ensure that the identified microbial clustering was driven by genuine structural shifts in community composition rather than being confounded by the unequal within-group dispersion common in microbiome datasets (31, 32).

#### NanoString data analysis

We used the ruv package (v0.9.7.1) in R (v4.1) to normalize the raw count and the batch effect for NanoString data. Pairwise comparison among experimental groups were conducted using the limma package (v3.58.1). Differentially expressed genes with FDR <0.05 were selected and used as inputs for gene set enrichment analysis (GSEA) (v4.2.3, Broad Institute), with the KEGG gene set used for enrichment score calculations.

#### Data analysis for all other endpoints

Differences in SCFA levels among groups were investigated by one-way ANOVA followed by Tukey’s multiple comparisons test. Differences in tumor growth/burden among experimental groups were analyzed by two-way repeated measures ANOVA followed by Student–Newman–Keuls post hoc test. Differences in E0771 mammary tumor “take” in mice allografted cells in both fourth mammary glands were analyzed by the chi-square test. The effects of genistein and tamoxifen treatment on E0771 cells in vitro were analyzed by two-way repeated measures ANOVA. Differences in tumor multiplicity, percentage of immune cells in the tumors, and expression of genes in Th17 pathway were analyzed by two-way ANOVA followed by Tukey’s multiple comparisons test. GraphPad Prism and R package agricolae (v1.3-7) were used to perform these statistical analyses. Results with p-values <0.05 were considered statistically significant.

## Results

### High- and low-MAC diets have differential effects on the gut microbiome

**Figure 2:**
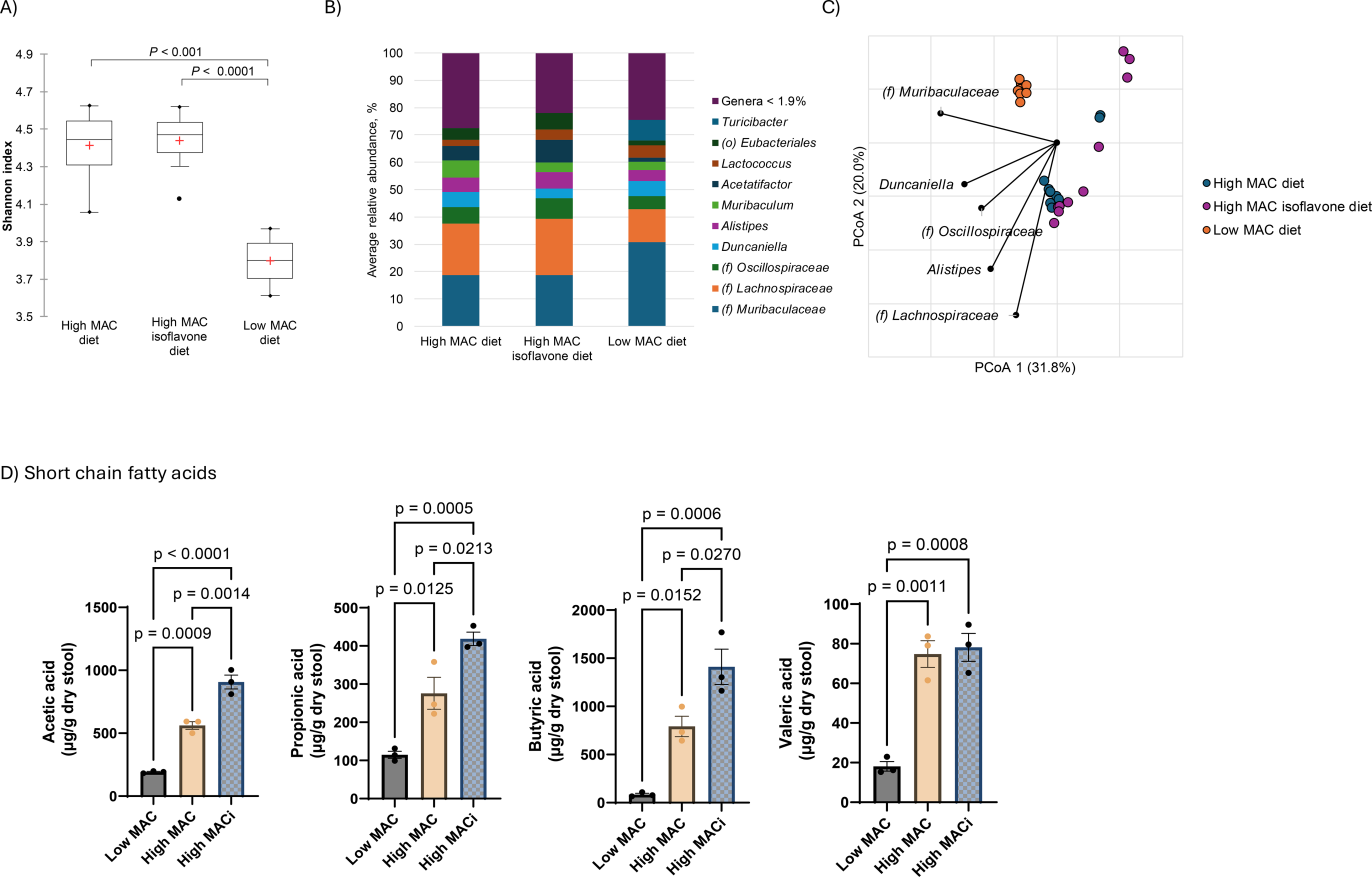
Microbiome diversity and composition and short-chain fatty acid (SCFA) levels in fecal samples of mice receiving different diets. In mice fed low-MAC, high-MAC, or high-MAC isoflavone (high-MACi) diet. **(A)** Box plots of Shannon diversity. **(B)** Distribution of predominant genera classified according to genus or the most resolved taxonomic level. (f) and (o) indicate taxa that could not be classified beyond the family or order, respectively. **(C)** Principal coordinate analysis of Bray–Curtis dissimilarities (r2 = 0.82). **(D)** Fecal SCFA levels. C57BL/6Tac mice were kept on one of the three diets for 4 weeks, after which fecal pellets were collected from 10 mice per group. Fecal pellets for SCFA assays were pooled to have three biological replicates per group. Data are shown as mean ± SEM with statistical significance obtained by one-way ANOVA.

Alpha diversity was significantly greater in mice fed high-MAC or high-MACi diet, compared with the low-MAC diet (**Figure 2A**). Communities were predominantly comprised of members of the SCFA-producing families Muribaculaceae, Lachnospiraceae, and Oscillospiraceae (**Table 1**; **Figure 2B**). Like alpha diversity, significant differences among relative abundances of most taxa were seen between the low- and the two high-MAC diets, with little distinction between the high-MAC and high-MACi diets (**Table 1**). *Muribaculum* of the Muribaculaceae family was the only genus that had significantly greater relative abundance, roughly twofold, in the high-MAC diet relative to both high-MACi and low-MAC diets. However, the Muribaculaceae family was significantly higher in the low-MAC than the two high-MAC diets. According to these findings, significantly higher abundances of the Muribacululaceae family and its *Muribaculum* genus was seen in mice that were responsive to anti-PD1. Community composition structure, as evaluated using Bray–Curtis dissimilarities, was significantly different among all groups (ANOSIM R = 0.53, *p* < 0.001; **Figure 2C**), which was supported by *post hoc* pairwise comparisons (*R* = 0.27–0.71, *p* ≤ 0.004, at Bonferroni-corrected *α* = 0.017).

**Table 1:** Distribution of predominant taxa under different diets.

Taxa	High-MAC diet	High-MACi diet	Low-MAC diet	P-value
(o) Eubacteriales	4.16 ± 0.96 A	6.19 ± 2.58 A	1.71 ± 0.31 B	<0.0001
(f) Lachnospiraceae (of the Eubacteriales order)	18.87 ± 3.47 A	20.57 ± 5.71 A	12.15 ± 2.58 B	<0.001
(f) Oscillospiraceae (of the Eubacteriales order)	6.04 ± 0.78	7.44 ± 3.59	4.88 ± 0.86	0.059
(f) Muribaculaceae	18.76 ± 5.81 B	18.75 ± 2.98 B	30.70 ± 4.16 A	<0.001
Muribaculum (of the Muribaculaceae family)	6.10 ± 2.40 A	3.45 ± 1.70 B	2.99 ± 0.37 B	0.001
Duncaniella (of the Muribaculaceae family)	5.48 ± 2.49	3.60 ± 1.63	5.36 ± 2.43	0.152
Alistipes	5.34 ± 1.48 AB	6.18 ± 1.47 A	4.02 ± 1.28 B	0.016
Acetatifactor	5.38 ± 2.72 A	8.26 ± 3.02 A	1.60 ± 0.32 B	<0.001
Lactococcus	2.32 ± 1.89 A	3.70 ± 1.58 A	4.50 ± 1.82 A	0.048
Turicibacter	0.01 ± 0.01 B	0.01 ± 0.01 B	7.64 ± 4.22 A	<0.0001

Taxa are classified according to genus or the most resolved taxonomic level. (f) and (o) indicate designations could only be made according to family and order, respectively.

### High-MAC diets increased fecal SCFA levels

Both high-MAC diets (high-MAC and high-MACi) significantly increased the concentrations of acetic acid (*p* = 0.009 and *p* < 0.0001), propionic acid (*p* = 0.01 and *p* = 0.0005), butyric acid (*p* = 0.015 and *p* = 0.0006), and valeric acid (*p* = 0.0011 and *p* = 0.0008) in stool, compared with the low-MAC diet (**Figure 2D**). The levels of acetic acid (*p* = 0.001), propionic acid (*p* = 0.02), and butyric acid (*p* = 0.02) were significantly higher in the high-MACi than in the high-MAC-fed mice (**Figure 2D**).

### Study 1

#### TNBC model

##### Effect of low- and high-MAC diets on TNBC growth and effectiveness of the anti-PD1 treatment

**Figure 3:**
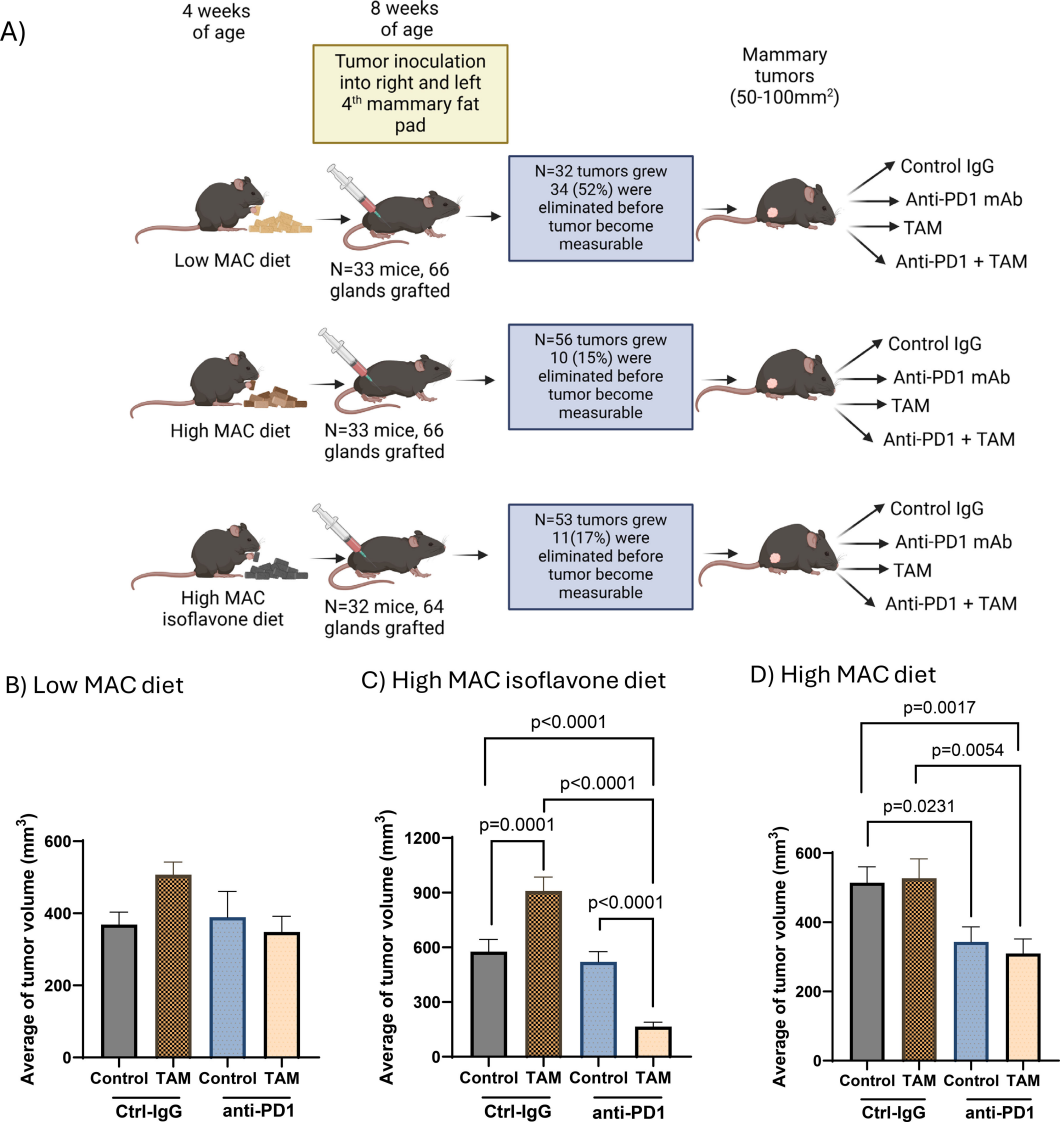
Effect of anti-PD1 and/or tamoxifen on the growth of E0771 TNBC in mice fed different diets. **(A)** Experimental design. **(B–D)** Tumor burden in mice treated with control IgG (Ctrl-IgG), tamoxifen (TAM), anti-PD1, or TAM+anti-PD1 and fed **(B)** low-MAC diet, **(C)** high-MAC isoflavone diet (high-MACi), or **(D)** high-MAC diet. Each mouse was allografted with two tumors: one on the right and another on the left fourth mammary gland. Tumor burden was calculated by combining the volume of the right and left tumors and then as an average of the measurement period per mouse, according to the R package agricolae (v1.3-7). There were four to eight mice in each group. Data are shown as mean ± SEM with statistical significance obtained by two-way repeated measures ANOVA followed by Student–Newman–Keuls post hoc test. Experimental design illustration was created with BioRender.com.

Fifty-two percent (52%) of the E0771 tumors allografted to the fourth mammary glands of both the right and left sides of a mouse that was fed the low-MAC diet did not grow in either mammary gland, i.e., tumor cells might have been cleared by the immune system (**Figure 3A**). Although tumors failed to grow in 15% of the allografted tumors in the high-MAC-fed mice and in 17% of the tumors allografted to the high-MACi-fed mice, these values were significantly lower than 52% in the low-MAC-diet-fed mice (*p* < 0.0001; chi-square test). If one tumor did not start to grow, the other in the same mouse did not grow either. Furthermore, two mammary tumors within the same mouse showed the same trend in tumor progression or regression, i.e., both “tumor-take” and the response to anti-PD1 were mouse-centric rather than tumor-centric, as also reported by others (33).

Anti-PD1 treatment had no effect on mammary tumor growth in animals fed either a low-MAC (**Figure 3B**) or high-MACi diet (**Figure 3C**). In the high-MAC group, a significant inhibition in tumor growth was observed by anti-PD1 therapy, compared with Ctrl-IgG (*p* = 0.023) (**Figure 3D**).

##### Effect of anti-estrogen tamoxifen on E0771 mammary tumor growth in mice fed low- or high-MAC diets

Since isoflavones bind and activate ERα (34–36), and activation of ERα activates immunosuppressive cells (10, 11), we questioned whether the lack of efficacy of anti-PD1 therapy in mice fed high-MACi diet might be due to the isoflavone genistein activating ERα in the TME. We explored this by treating mice with TAM, a partial ERα antagonist. TAM has been reported to block ERα in the tumor and increase infiltration of CD4^+^ T cells (37) and activate CD8^+^ T cells (38) in the TME. In breast cancer patients, TAM suppressed myeloid-derived suppressor cells (MDSCs) (39). Estradiol is reported to have an opposite effect on MDSCs (10, 11). In this study, mice were allografted E0771 mammary tumors to both the left and right fourth mammary glands. Among the low-MAC- and high-MAC-diet-fed mice, tumor burden was similar in the Ctrl-IgG and TAM-treated mice (**Figures 3B, D**). Unexpectedly, mice fed the high-MACi diet exhibited increased E0771 tumor growth when treated with TAM, compared with Ctrl-IgG-treated mice (*p* = 0.0001) (**Figure 3C**).

##### Effect of diet, tamoxifen, and anti-PD1 treatment on tumor growth

Treatment with a combination of anti-PD1 plus TAM significantly inhibited mammary tumor growth in mice fed the high-MACi diet, compared with TAM or anti-PD1 treatments as monotherapies or with Ctrl-IgG (*p* < 0.0001) (**Figure 3C**). The combination treatment did not further reduce tumor growth in the high-MAC-diet-fed mice, compared with anti-PD1 treatment alone (**Figure 3D**), nor did it affect tumor growth in the low-MAC-diet-fed mice (**Figure 3B**). Thus, although TAM on its own promoted tumor growth in the high-MACi group, in combination with anti-PD1, it induced highly significant tumor growth reduction. These data suggest that blocking ERα in the TME of TNBC is required for anti-PD1 to be effective in an elevated estrogenic environment.

#### ERα^+^ breast cancer model

##### Effect of low- and high-MAC diets on ERα*^+^* mammary tumor growth and anti-PD1 response

**Figure 4:**
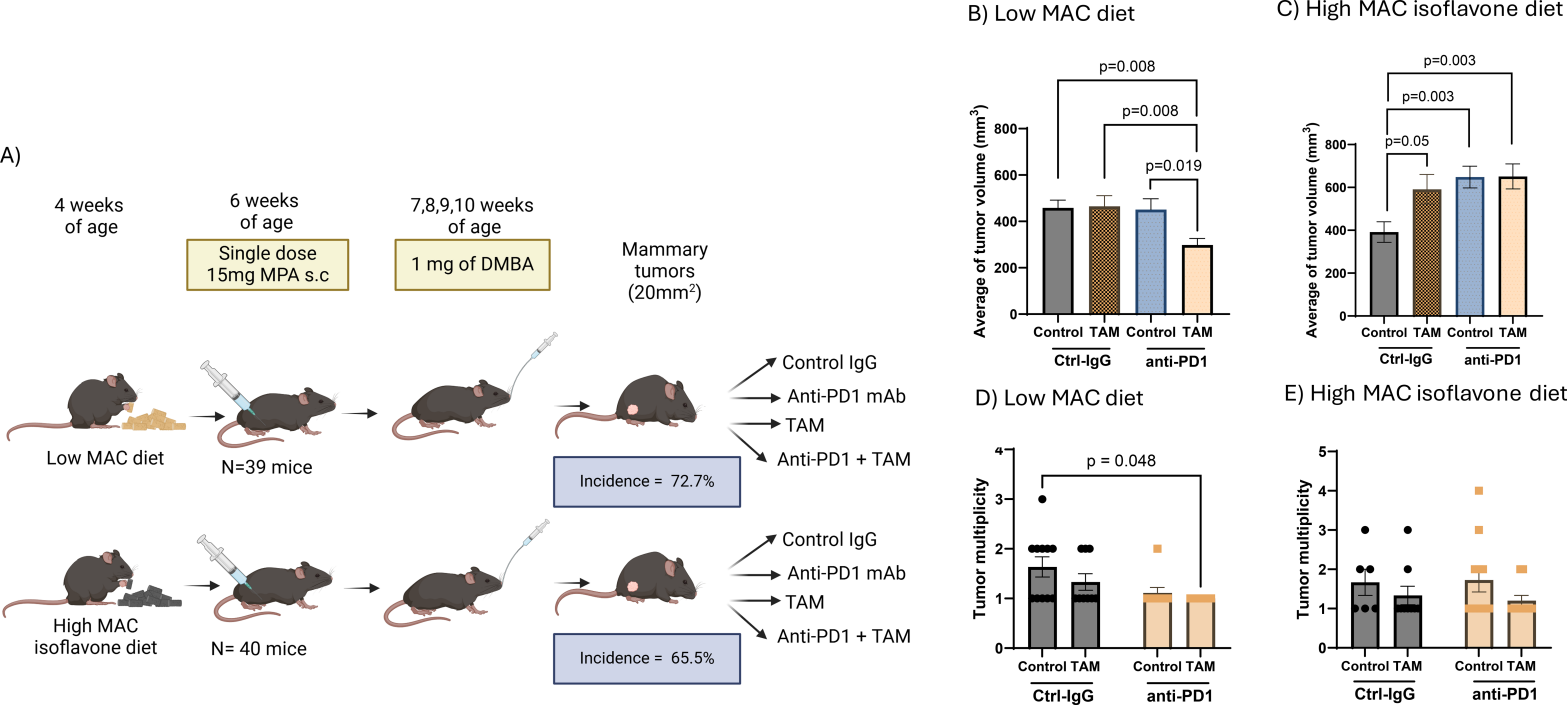
Effect of anti-PD1 and/or tamoxifen on the growth of ERα+ tumors in mice fed different diets. **(A)** Experimental design of 7,12-dimethylbenz[a] anthracene (DMBA)-initiated estrogen receptor α-positive (ERα+) mammary tumors in mice fed low-MAC or high-MAC isoflavone (high-MACi) diet. **(B, C)** Tumor burden in mice treated with Ctrl-IgG, TAM, anti-PD1, or TAM+anti-PD1 and fed **(B)** low-MAC diet or **(C)** high-MACi diet. Tumor burden per mouse was calculated by combining the volume of all tumors and then as an average of the measurement period per mouse, according to the R package agricolae (v1.3-7). Tumor multiplicity in mice treated with Ctrl-IgG, TAM, anti-PD1, or TAM+anti-PD1 and fed **(D)** low MAC diet or **(E)** high-MACi diet. There were 7–11 mice in each group. Data are shown as mean ± SEM with statistical significance obtained by two-way repeated measures ANOVA followed by Student–Newman–Keuls post hoc test for tumor burden and two-way ANOVA followed by Tukey’s multiple comparisons test for tumor multiplicity. Experimental design illustration was created with BioRender.com.

We compared the effect of low-MAC and high-MACi diets on anti-PD1 responsiveness in the ERα^+^ DMBA model. The experimental design of the study is shown in **Figure 4A**. The incidence of DMBA-induced tumors was similar in the two dietary groups (**Figure 4A**). Among mice that were fed the high-MACi diet, 65.5% of mice developed mammary tumors (multiplicity 1.5 ± 0.13 per mouse), and among mice fed the low-MAC diet, 72.7% of mice developed mammary tumors (multiplicity 1.3 ± 0.09 per mouse). Thus, diet did not affect the incidence or multiplicity of mammary tumors. In the low-MAC diet group, anti-PD1 did not impact tumor growth (**Figure 4B**). When compared with Ctrl-IgG, anti-PD1 therapy in mice fed the high-MACi diet unexpectedly significantly promoted tumor growth (*p* = 0.003) (**Figure 4C**).

##### Effect of diet and tamoxifen on tumor growth

TAM stimulated DMBA-induced mammary tumor growth in mice fed a high-MACi diet (*p* = 0.05) (**Figure 4C**) but did not affect tumor growth in mice fed a low-MAC diet (**Figure 4B**). These results are like those observed in mice allografted with E0771 TNBC, i.e., TAM increased tumor growth when given an isoflavone-containing diet, regardless of whether the tumors expressed ERα. These findings suggest that the tumor growth-potentiating effects of TAM in mice fed the high-MACi diet were not linked to ERα status of the tumors but to the effects of isoflavones on the TME.

##### Effect of diet, tamoxifen, and anti-PD1 treatment on tumor growth

The combination treatment with TAM and anti-PD1 inhibited tumor growth in the low-MAC-fed mice with ERα^+^ mammary tumors compared with Ctrl-IgG (*p* = 0.008), anti-PD1 (*p* = 0.019), or TAM (*p* = 0.008) monotherapy-treated mice (**Figure 4B**). The combination treatment also decreased tumor multiplicity compared to mice treated with Ctrl-IgG (*p* = 0.048) in mice fed the low-MAC diet (**Figure 4D**). In contrast, in the high-MACi group, tumor growth during combination treatment was like that seen in mice treated with TAM or anti-PD1 alone (**Figure 4C**). There was no difference in multiplicity among mice fed the high-MACi diet (**Figure 4E**). These findings are opposite to the results obtained in mammary tumors from mice allografted with E0771 cells. In the TNBC model, TAM+anti-PD1 effectively reduced E0771 tumor growth when mice were fed the high-MACi diet. Thus, the presence or absence of ERα in mammary tumors might impact how diet and TAM modify the response to anti-PD1 therapy.

### Study 2

#### Allografting E0771 cells to a single mammary gland per mouse

In this experiment, each mouse was allografted E0771 tumor cells into only the right fourth mammary gland, resulting in 100% of allografted tumors to start growing in the low-MAC-, high-MAC-, and high-MACi-diet-fed mice. Anti-PD1 was now effective in inhibiting tumor growth both in mice that were fed a low-MAC (*p* = 0.0009) (**Figure 1A**) and a high-MAC diet (*p* = 0.0182) (**Figure 1B**). However, like in study 1 (**Figure 3C**), mice fed the high-MACi diet did not respond to anti-PD1 therapy (**Figure 1C**).

### Study 3

#### Effect of the isoflavone genistein on anti-PD1 response

In mice with a single E0771 tumor allograft and fed low-MAC diet, anti-PD1 at doses of 100 μg or 150 μg significantly reduced tumor burden compared with Ctrl-IgG (*p* < 0.0001) (**Figures 1D, E**). Adding genistein to the low-MAC diet significantly reduced the effect of anti-PD1 on tumor growth (*p* = 0.0202 and *p* < 0.0001 between anti-PD1-control and anti-PD1-genistein mice for the first replicate and second replicate, respectively) (**Figures 1D, E**).

#### Effect of tamoxifen on genistein-treated E0771 mammary tumor cells *in vitro*

E0771 mammary tumor cells did not express ERα protein but were positive for ERβ (**Figure 1F**). Consistent with these cells being ERα negative, 1 μM of TAM did not affect their growth (**Figure 1G**). However, 5 µM of genistein significantly inhibited E0771 tumor cell proliferation (*p* = 0.016) (**Figure 1G**). The combination of TAM + genistein did not further inhibit growth, i.e., TAM did not add to the growth inhibitory effect of genistein. Since genistein binds to cancer growth suppressing ERβ (40), these findings suggest that activation of ERβ by genistein in tumor cells, in the absence of ERα, can directly inhibit the growth of E0771 mammary tumors. The data also indicate that TAM’s E0771 tumor growth-promoting effect *in vivo* in high-MACi-fed mice likely was caused by the combined effect of TAM and isoflavones on the TME.

#### Effect of low- and high-MAC diets on tumor CD8^+^ T-cell infiltration and exhaustion in anti-PD1-treated mice

**Figure 5:**
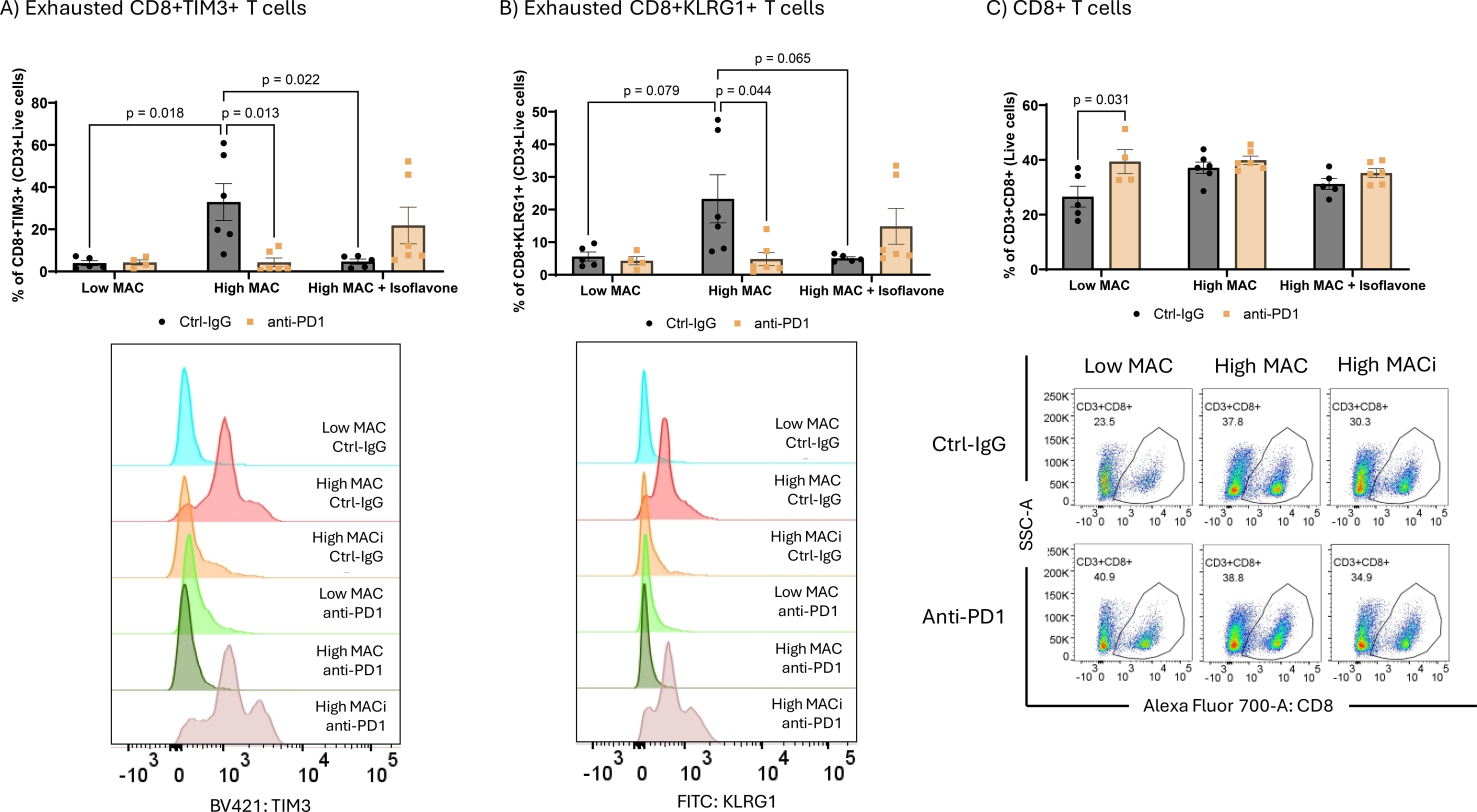
Effect of anti-PD1 on CD8+ T-cell exhaustion in mice fed different diets. Frequencies of **(A)** CD8+TIM3+ cells (CD8+TIM3+), **(B)** CD8+KLRG1+ cells, and **(C)** total CD8+ cells (CD3+CD8+) in tumors of mice fed low-MAC, high-MAC, or high-MAC isoflavone (high-MACi) diets and treated with anti-PD1 or Ctrl-IgG. Cells were gated on live cells, followed by CD3+ cells, CD8+ cells, and TIM3+ or KLRG1+ cells. Gates were determined based on FMO staining. There were four to six tumors in each group. Data are shown as mean ± SEM with statistical significance obtained by two-way ANOVA followed by Tukey’s multiple comparisons test.

The high-MAC-diet-fed mice exhibited a significantly higher proportion of exhausted TIM3^+^CD8^+^ cells in the TME than mice fed a low-MAC diet (*p* = 0.018) or a high-MACi diet (*p* = 0.022) (**Figure 5A**). These findings were supported by the exhaustion marker KLRG1; specifically, the high-MAC group exhibited a trend toward higher CD8^+^KLRG1^+^ cell frequency than the low-MAC (*p* = 0.079) and high-MACi groups (*p* = 0.065) (**Figure 5B**). Infiltration of CD8^+^ T cells was similar among the three dietary groups (**Figure 5C**). Treatment with anti-PD1 significantly suppressed the CD8^+^TIM3^+^ and CD8^+^KLRG1^+^ T-cell populations in the TME of high-MAC-diet-fed mice (*p* = 0.013 and *p* = 0.044, respectively) but increased these cells in the high-MACi group (*p* = 0.002 and *p* = 0.01 for interaction between diet and anti-PD1). However, no significant differences were observed in the frequency of CD8^+^PD1^+^ T cells across the various diet or treatment groups (**Supplementary Figure 1**). Furthermore, anti-PD1 increased CD8^+^ T-cell infiltration in the low-MAC diet group (*p* = 0.031) (**Figure 5C**), indicating a stronger antitumor immune response. The findings obtained here are consistent with mice fed the high-MAC diet being responsive to anti-PD1 therapy and the high-MACi-diet-fed mice being resistant.

### NanoString analysis in the high-MACi-diet-fed mice

Since TAM sensitized E0771 tumors in mice fed the high-MACi diet to anti-PD1, a NanoString analysis with immune gene platform was performed using mammary tumors obtained from study 1 from mice fed the high-MACi diet and treated with either Ctrl-IgG, anti-PD1, TAM, or TAM+anti-PD1. A total of 123 genes were differentially expressed in mice fed the high-MACi diet and treated with TAM+anti-PD1 versus those treated with Ctrl-IgG, anti-PD1, or TAM monotherapy (**Supplementary Table 3**). In the KEGG analysis of these genes, several pathways were identified that were significantly different in the TAM+anti-PD1 group, compared with the three other treatment groups. Of these, excluding any disease-specific pathways, we focused on 12 relevant pathways in which more than 10% of pathway genes were differentially expressed. The highest percentile of differentially expressed genes (17.4%) belonged to the Th17 cell differentiation pathway. Others were Toll-like receptor signaling, NF-κB signaling, PDL1 expression and PD1 checkpoint in cancer, AGE-RAGE signaling, JAK-STAT signaling, Th1 and Th2 differentiation, IL-17 signaling, HIF-1 signaling, TNF signaling, cytokine–cytokine receptor interaction, and C-type lectin receptor signaling pathways, in the order of highest to lowest percentile of genes involved per pathway (**Table 2**).

**Table 2:** Signaling pathways identified in tumors of high-MACi mice treated with TAM+anti-PD1, compared with Ctrl-IgG, TAM, or anti-PD1.

Pathway	% altered genes of the total genes in the pathway
TH17 cell differentiation	17.4%
Toll-like receptor signaling	16.5%
NF-κB signaling	16.2%
PDL1 expression and PD1 checkpoint in cancer	15.6%
AGE-RAGE signaling in diabetic conditions	13.9%
JAK-STAT signaling	13.1%
Th1 and Th2 differentiation	12.9%
IL-17 signaling	11.6%
HIF-1 signaling	11.0%
TNF signaling	10.9%
Cytokine–cytokine receptor interaction	10.7%
C-type lectin receptor signaling	10.6%

### Confirming gene expression changes in mammary tumors

**Figure 6:**
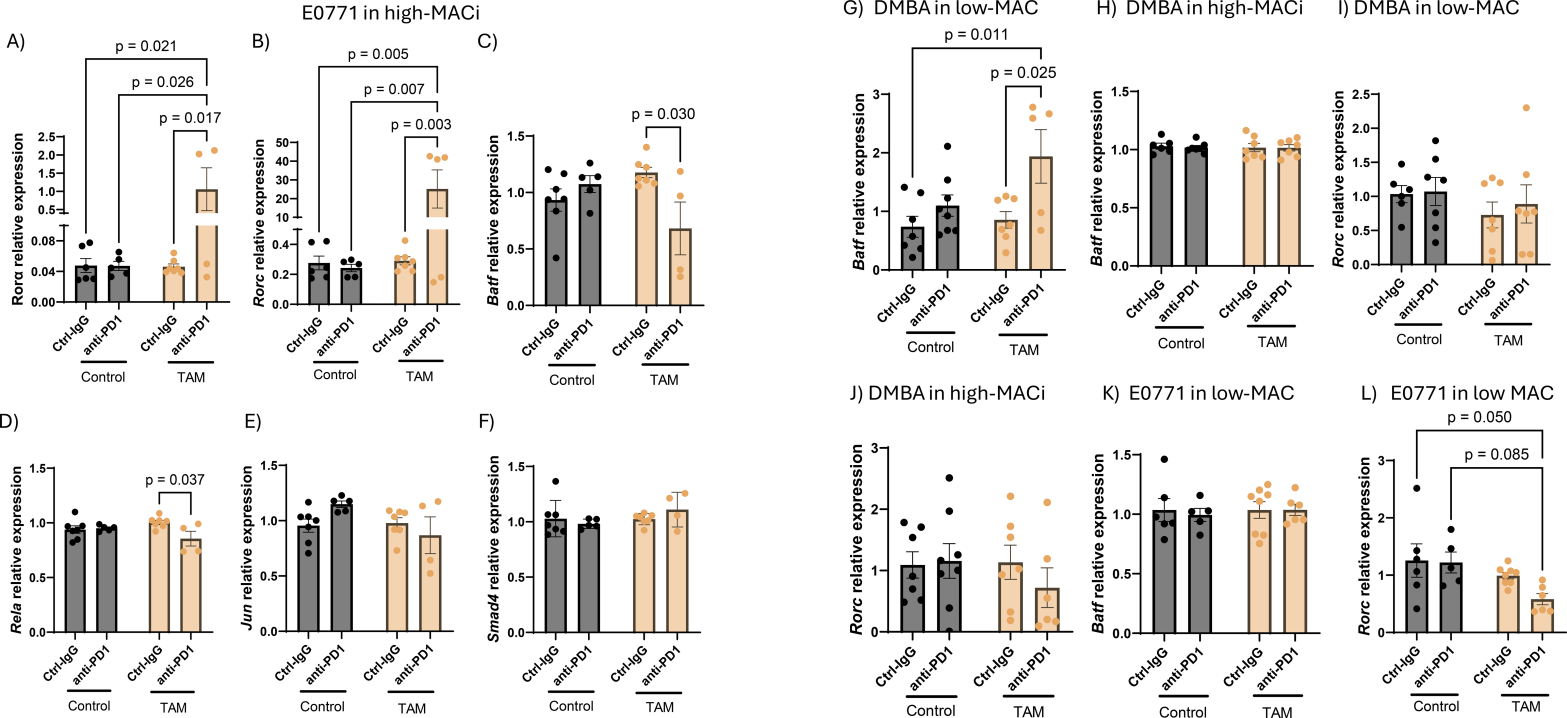
Confirmation of expression of genes in the Th17 differentiation pathway. Expression of **(A)** Rorα, **(B)** Rorc, **(C)** Batf, **(D)** Rela, **(E)** Jun, and **(F)** Smad4 in mammary tumors of mice fed high-MAC isoflavone (high-MACi) diet and treated with Ctrl-IgG, anti-PD1, tamoxifen (TAM), or TAM+anti-PD1. Expression of **(G, H)** Batf and **(I, J)** Rorc in 7,12-dimethylenz[a]anthracene (DMBA)-initiated estrogen receptor α-positive (ERα+) mammary tumors in low-MAC or high-MACi diet, or **(K)** Batf and **(L)** Rorc in E0771 TNBCs in low-MAC-fed mice. Mice were treated with Ctrl-IgG, anti-PD1, tamoxifen (TAM), or TAM+anti-PD1. There were four to eight tumors in each group. Data are shown as mean ± SEM with statistical significance obtained by two-way ANOVA followed by Tukey’s multiple comparisons test.

To confirm differential gene expression within the Th17 differentiation pathway in E0771 tumors from mice fed the high-MACi diet, we focused on *Batf*, *Jun*, *Rela*, *Rorα*, *Rorc*, and *Smad4*. Of these, in the TAM+anti-PD1 combination treatment group, *Rorα* and *Rorc* were upregulated compared with all the other treatment groups (for *p*-values, see **Figures 6A,B**), and *Batf* (*p* = 0.030) (**Figure 6C**) and *Rela* (*p* = 0.037) (**Figure 6D**) were suppressed compared with TAM. These findings are consistent with the data obtained by NanoString. Changes in *Jun* or *Smad4* could not be confirmed (**Figures 6E, F**).

We next investigated whether the Th17 differentiation pathway genes *Batf* and *Rorc* were changed also in ERα^+^ mammary tumors in mice fed the low-MAC diet, since combination treatment with TAM+anti-PD1 converted ICB unresponsive ERα^+^ tumors responsive in mice fed this diet. *Batf* was significantly upregulated in the low-MAC-diet-fed mice treated with TAM+anti-PD1 compared with untreated control mice (*p* = 0.011) or TAM (*p* = 0.025)-treated mice (**Figure 6G**), but there were no differences among the groups of mice fed the high-MACi diet (**Figure 6H**). The expression of *Rorc* among mice treated with Ctrl-IgG, anti-PD1, TAM, or their combination in the ERα^+^ model in mice fed the low-MAC or high-MACi diet was not affected either (**Figures 6I, J**). In the TNBC model in mice fed the low-MAC diet, *Batf* expression was not altered among the four treatment groups (**Figure 6K**), but combination treatment (TAM+anti-PD1) reduced *Rorc*, compared with the Ctrl-IgG-treated mice (*p* = 0.05) (**Figure 6L**). Thus, upregulation of *Rorα* and *Rorc* and downregulation of *Batf* and *Rela* may be linked to the reversal of ICB resistance in the TNBC model, but not in the ERα^+^ model. In the ERα^+^ mammary tumors, upregulation of *Batf* was associated with the response to anti-PD1 therapy under the low-MAC diet and co-treatment with TAM. Taken together, our findings indicate that although the Th17 differentiation pathway might be related to improved anti-PD1 response in TAM-treated mice, individual genes in this pathway driving responsiveness seem to be different in the TNBC and ERα^+^ mammary tumor models.

## Discussion

The gut microbiome plays an important yet not clearly defined role in determining the effectiveness of ICB (41, 42). Diet, especially fiber, modifies the gut microbiome (43), but studies have not consistently shown that fiber intake promotes response to ICBs (5). We studied here whether the effectiveness of anti-PD1 therapy in TNBC and ERα^+^ mammary tumor models that have not been included into previous studies investigating connections between dietary fiber and ICB response might be different depending on whether mice are fed a low- or high-MAC diet. The diets in our study contained different amounts of isoflavones. Some evidence indicates that fiber improves the response to ICB therapy (1), while isoflavones may either improve or impair the response (9). Our results indicated that both low- and high-MAC-diet-fed mice responded to anti-PD1 therapy, but if mice were fed the high-MAC diet containing notable levels of isoflavones, they did not.

Analysis of fecal SCFA levels showed that high-MAC and high-MACi diets significantly increased fecal acetic, butyric, propionic, and valeric acids compared with the low-MAC diet, with the high-MACi diet resulting in the highest increases (3–10-fold higher SCFAs). Other studies have found an increase in fecal SCFAs in mice fed the high-MAC diet, compared with the low-MAC diet like the one used in our study (44, 45). These results suggest that elevated fecal SCFA levels alone do not determine responsiveness to cancer immunotherapies. However, analyzing plasma SCFA levels is critical, as they may more accurately reflect the systemic effects of SCFAs and their subsequent impact on mammary tumor growth (46). Furthermore, while the SCFA results yielded highly significant differences despite the low biological replicate number, these specific metabolic profiles should be interpreted as exploratory findings that warrant confirmation in future, larger-scale validation studies.

At the gut microbial level, both the high-MAC and high-MACi diets elevated alpha diversity and the abundance of the SCFA-producing bacterial families Lachnospiraceae and Oscillospiraceae, compared with the low-MAC diet. The low-MAC diet elevated the abundance of the SCFA-producing Muribaculaceae, and the high-MAC diet, but not the high-MACi diet, increased the abundance of the *Muribaculum* genus of the Muribaculaceae family. These results suggest that diets, which resulted in responsiveness to anti-PD1 therapy, elevated either fecal Muribaculaceae or *Muribaculum*. Similar data were generated in a human study, which showed that high fecal abundance of *Muribaculum* correlated with increased effectiveness of cancer immunotherapy (47).

Because different experimental cohorts were evaluated across two distinct animal facilities, we acknowledge that cross-institutional variations in animal husbandry represent a potential limitation that alters baseline gut microbiota composition. However, prior work from our group utilizing cross-institutional cohorts confirmed that while baseline gut microbiome drift occurs, the main directional shifts in microbial composition and specific biomarkers (e.g., *Akkermansia* depletion) induced by our target interventions remain stable and reproducible across distinct facilities (48).

Since the high-MACi-diet-fed mice, which had the highest fecal SCFA levels, did not respond to anti-PD1 monotherapy, we studied whether isoflavones might blunt the response to anti-PD1. The isoflavone genistein binds and activates ERα and ERβ (34), exhibiting stronger activity toward ERβ than ERα (36), and breast cancer cells that express higher levels of ERβ than ERα are inhibited by genistein (34). Genistein did not directly stimulate the growth of murine E0771 cells *in vitro*, consistent with E0771 mammary tumor cells expressing ERβ but not ERα (49), as also confirmed here. In immune cells, activation of ERα induces immunosuppression, while activation of ERβ stimulates antitumor immunity (50). However, since T cells in the TME express higher levels of ERα than ERβ (51), genistein may have promoted immunosuppression, explaining the failure of E0771 tumors to respond to anti-PD1 *in vivo* when mice were fed the high-MACi diet or the low-MAC diet supplemented with genistein.

The ERα antagonist TAM dramatically improved anti-PD1 responsiveness in mice fed the high-MACi diet, possibly because TAM has been reported to activate CD8^+^ T cells (38) in the TME. In earlier studies, another ERα antagonist (fulvestrant) improved response to ICBs in estrogen-insensitive and ICB-resistant preclinical non-breast cancer models by inhibiting immunosuppressive MDSCs (10, 15). We did not assess the effects of genistein on immune cells but only focused on CD8^+^ T-cell activation and exhaustion in mice fed the low- or high-MAC/MACi diets. In human studies, when breast cancer TME contains high levels of exhausted CD8^+^ T cells, patients are most responsive to ICB (52, 53). In our study, CD8^+^ T-cell infiltration into E0771 TME was similar in the high-MAC- and high-MACi-diet-fed mice, but non-significantly lower in the low-MAC-diet-fed mice. However, the percentage of exhausted CD8^+^ T cells was the highest among mice fed the high-MAC diet, and treatment with anti-PD1 significantly reduced an exhaustion marker in these cells. Anti-PD1 treatment tended to have an opposite effect on high-MACi-diet-fed mice, consistent with the high-MACi-diet-fed mice not responding to anti-PD1 monotherapy. Our results thus agree with exhausted CD8^+^ T cells being a marker of responsiveness to ICBs in TNBC. Among the low-MAC diet fed mice which exhibited very low levels of exhausted CD8^+^ T cells but also responded to anti-PD1, anti-PD1 treatment increased total CD8^+^ T-cell infiltration into the TME. Future studies need to address in more detail the effect of genistein, alone or in combination with TAM, on immune cells in the TME.

Like ERα^+^ breast cancers in women (54) and animal models (26), ERα^+^ DMBA mammary tumors in our study were resistant to ICB. However, DMBA tumors became responsive to anti-PD1 treatment if mice were fed isoflavone-free low-MAC diet and co-treated with TAM. Thus, the presence of isoflavones in the high-MAC diet appeared to have blocked the effects of combination treatment with anti-PD1 and TAM that was observed when mice were fed the low-MAC diet. It is important to acknowledge the differences between the models used in this study. The E0771 mammary tumors represent a model in which existing tumor cells are grafted into the mammary fat pad of a syngeneic mice, while the DMBA ER^+^ model, involves carcinogen-induced initiation of *de novo* tumor development. These differences could have influenced the effectiveness of diet, immunotherapy, and endocrine therapy combinations. Whether patients with advanced ERα^+^ breast cancer might be more likely to respond to anti-PD1 therapy if they consume diets low in estrogens should be studied. High estrogenic foods are those that elevate circulating estrogen levels or that contain estrogenic compounds, like isoflavones. Foods that elevate circulating estrogen levels have been linked to increased breast cancer risk (55, 56), but their possible association to responsiveness to ICBs has not been investigated.

Another potential limitation of this study is the variation in anti-PD1 dosages across the different experimental cohorts. However, these doses were selected based on the known baseline therapeutic responses of each specific model. For the DMBA-induced model, a higher (200 µg) anti-PD1 dose was warranted given the baseline immunotherapy resistance commonly observed in carcinogen-driven tumors (25). Conversely, modulating the E0771 models with down-titrated amounts optimized our experimental capacity to reveal subtle, complex dietary microbiome or hormonal cross-talk without creating an artificial therapeutic ceiling. Importantly, the finding that anti-PD1 therapy generated consistent responses across these customized variations further validates the core translational conclusions of the study.

To study the interaction between TAM and anti-PD1 in gene signaling level, we performed NanoString analysis using the cancer immune profiling platform. The Th17 cell differentiation pathway was the top immune signaling pathway that differed significantly in mice fed a high-MACi diet and treated with a combination of TAM+anti-PD1, compared with the other three treatment groups. The role of Th17 cells in carcinogenesis has been conflicting (57). The controversy might be caused by the identification of two subtypes of Th17 cells: pathogenic and non-pathogenic (58). In our study, *Rorα* and *Rorc* were upregulated and *Batf* and *Rela* were downregulated in mice the fed high-MACi diet and treated with TAM+anti-PD1. Upregulated *RorA*, which is activated downstream of IL-6 to induce differentiation of Th17 cells (59), can act as a tumor suppressor gene (60) and is anti-inflammatory by inhibiting NF-κB (61). The other upregulated gene plays a key role downstream of IL-6 and TGFβ and works synergistically with *Rorα* to induce lineage specification of uncommitted CD4^+^ T cells into Th17 cells (62). *Rorc* also improves responsiveness to cancer immunotherapy, including anti-PD1 treatment (63). *Rela* promotes cancer cell growth and is involved in the polarization of uncommitted CD4^+^ T cells towards pathogenic Th17 cells (64). *Batf* also participates in maintaining the differentiation and function of Th17 cells (65). Downregulation of *Rela* and *Batf* in the high-MACi-fed mice treated with TAM+anti-PD1 is thus consistent with the inhibition of E0771 tumor growth.

Expression of *Rorc* was not affected in ERα^+^ mammary tumors by diet or anti-PD1, TAM, or TAM+anti-PD1 treatments. Instead, *Batf* was upregulated in low-MAC-diet-fed mice treated with TAM+anti-PD1, which consequently exhibited reduced tumor growth. These results suggest that in the ERα^+^ mammary tumor model, *Batf* might have functioned to improve responsiveness to ICB treatment, as previously reported (66). In summary, the combination treatment might have activated non-pathogenic Th17 differentiation in mice fed the high-MACi diet.

Clinical trials in breast cancer patients are ongoing to determine the potential role of endocrine therapies in improving responsiveness to ICBs (67, 68). Whether the diet which these patients consume influences as to who benefits from the combination treatment has not been investigated. Our study highlights the importance of diet in determining ICB responsiveness in preclinical animal models. The results rebuff the idea that high-MAC diets, which elevate fecal SCFA levels, always generate a better response to anti-PD1. Rather, the results indicate that an even more important factor might be whether diet contains estrogenic components, such as isoflavones. Our findings show that isoflavones suppress response to anti-PD1, regardless of fecal SCFA levels, and blocking ERα^+^ in TNBC, i.e., probably in the tumor microenvironment with TAM, reversed the suppression. It is therefore crucial that when researchers are designing preclinical models to study cancer therapeutic response, they must consider the constituents of the laboratory diet of their animals and then report which lab chow was used. This will allow for accurate interpretation of results and ensure that the design of clinical trials based on these studies is appropriate. Our data also further highlight that human studies assessing the effectiveness of various combination therapies which include ICBs may need to consider interindividual variation in patients’ diet.

## Data Availability

The data presented in the study are deposited in the NCBI SRA under BioProject repository, accession number SRP579135 (https://www.ncbi.nlm.nih.gov/Traces/study/?acc=SRP579135&o=acc_s%3Aa).

## References

[1] Spencer CN McQuade JL Gopalakrishnan V McCulloch JA Vetizou M Cogdill AP . Dietary fiber and probiotics influence the gut microbiome and melanoma immunotherapy response. Science. (2021) 374:1632–40. doi: 10.1126/science.aaz7015 34941392 PMC8970537

[2] So D Whelan K Rossi M Morrison M Holtmann G Kelly JT . Dietary fiber intervention on gut microbiota composition in healthy adults: a systematic review and meta-analysis. Am J Clin Nutr. (2018) 107:965–83. doi: 10.1093/ajcn/nqy041 29757343

[3] Nomura M Nagatomo R Doi K Shimizu J Baba K Saito T . Association of short-chain fatty acids in the gut microbiome with clinical response to treatment with nivolumab or pembrolizumab in patients with solid cancer tumors. JAMA Netw Open. (2020) 3:e202895. doi: 10.1001/jamanetworkopen.2020.2895 32297948 PMC7163404

[4] Luu M Riester Z Baldrich A Reichardt N Yuille S Busetti A . Microbial short-chain fatty acids modulate CD8+ T cell responses and improve adoptive immunotherapy for cancer. Nat Commun. (2021) 12:4077. doi: 10.1038/s41467-021-24331-1 34210970 PMC8249424

[5] Roichman A Reyes-Castellanos G Chen Z Chen Z Mitchell SJ MacArthur MR . Dietary fiber lacks a consistent effect on immune checkpoint blockade efficacy across diverse murine tumor models. Cancer Res. (2025) 85:3335–47. doi: 10.1158/0008-5472.can-24-4378 40540354 PMC12402783

[6] Sonnenburg ED Sonnenburg JL . Starving our microbial self: the deleterious consequences of a diet deficient in microbiota-accessible carbohydrates. Cell Metab. (2014) 20:779–86. doi: 10.1016/j.cmet.2014.07.003 25156449 PMC4896489

[7] Koh A De VF Kovatcheva-Datchary P Backhed F . From dietary fiber to host physiology: short-chain fatty acids as key bacterial metabolites. Cell. (2016) 165:1332–45. doi: 10.1016/j.cell.2016.05.041 27259147

[8] Brown NM Setchell KD . Animal models impacted by phytoestrogens in commercial chow: implications for pathways influenced by hormones. Lab Invest. (2001) 81:735–47. doi: 10.1038/labinvest.3780282 11351045

[9] Focaccetti C Izzi V Benvenuto M Fazi S Ciuffa S Giganti MG . Polyphenols as immunomodulatory compounds in the tumor microenvironment: friends or foes? Int J Mol Sci. (2019) 20. doi: 10.3390/ijms20071714 30959898 PMC6479528

[10] Svoronos N Perales-Puchalt A Allegrezza MJ Rutkowski MR Payne KK Tesone AJ . Tumor cell-independent estrogen signaling drives disease progression through mobilization of myeloid-derived suppressor cells. Cancer Discov. (2017) 7:72–85. doi: 10.1158/2159-8290.cd-16-0502 27694385 PMC5222699

[11] Chakraborty B Byemerwa J Shepherd J Haines CN Baldi R Gong W . Inhibition of estrogen signaling in myeloid cells increases tumor immunity in melanoma. J Clin Invest. (2021) 131:e151347. doi: 10.1172/jci151347 34637400 PMC8631601

[12] Milette S Hashimoto M Perrino S Qi S Chen M Ham B . Sexual dimorphism and the role of estrogen in the immune microenvironment of liver metastases. Nat Commun. (2019) 10:5745. doi: 10.1038/s41467-019-13571-x 31848339 PMC6917725

[13] Tai P Wang J Jin H Song X Yan J Kang Y . Induction of regulatory T cells by physiological level estrogen. J Cell Physiol. (2008) 214:456–64. doi: 10.1002/jcp.21221 17654501

[14] Ye Y Jing Y Li L Mills GB Diao L Liu H . Sex-associated molecular differences for cancer immunotherapy. Nat Commun. (2020) 11:1779. doi: 10.1038/s41467-020-15679-x 32286310 PMC7156379

[15] Marquez-Garban DC Deng G Comin-Anduix B Garcia AJ Xing Y Chen HW . Antiestrogens in combination with immune checkpoint inhibitors in breast cancer immunotherapy. J Steroid Biochem Mol Biol. (2019) 193:105415. doi: 10.1016/j.jsbmb.2019.105415 31226312 PMC6903431

[16] Jensen MN Ritskes-Hoitinga M . How isoflavone levels in common rodent diets can interfere with the value of animal models and with experimental results. Lab Anim. (2007) 41:1–18. doi: 10.1258/002367707779399428 17234046

[17] Gohl DM Vangay P Garbe J MacLean A Hauge A Becker A . Systematic improvement of amplicon marker gene methods for increased accuracy in microbiome studies. Nat Biotechnol. (2016) 34:942–9. doi: 10.1038/nbt.3601 27454739

[18] Schloss PD Westcott SL Ryabin T Hall JR Hartmann M Hollister EB . Introducing mothur: open-source, platform-independent, community-supported software for describing and comparing microbial communities. Appl Environ Microbiol. (2009) 75:7537–41. doi: 10.1128/aem.01541-09 19801464 PMC2786419

[19] Staley C Kaiser T Vaughn BP Graiziger CT Hamilton MJ Rehman TU . Predicting recurrence of Clostridium difficile infection following encapsulated fecal microbiota transplantation. Microbiome. (2018) 6:166. doi: 10.1186/s40168-018-0549-6 30227892 PMC6145197

[20] Garcia-Villalba R Gimenez-Bastida JA Garcia-Conesa MT Tomas-Barberan FA Carlos Espin J Larrosa M . Alternative method for gas chromatography-mass spectrometry analysis of short-chain fatty acids in faecal samples. J Sep Sci. (2012) 35:1906–13. doi: 10.1002/jssc.2842 22865755

[21] Adams KJ Pratt B Bose N Dubois LG St John-Williams L Perrott KM . Skyline for small molecules: a unifying software package for quantitative metabolomics. J Proteome Res. (2020) 19:1447–58. doi: 10.1021/acs.jproteome.9b00640 31984744 PMC7127945

[22] Loveland BE McKenzie IF . Cells mediating graft rejection in the mouse. II. The Ly phenotypes of cells producing tumor allograft rejection. Transplantation. (1982) 33:174–80. doi: 10.1097/00007890-198202000-00013 7036470

[23] Sagiv-Barfi I Czerwinski DK Levy S Alam IS Mayer AT Gambhir SS . Eradication of spontaneous Malignancy by local immunotherapy. Sci Transl Med. (2018) 10. doi: 10.1126/scitranslmed.aan4488 29386357 PMC5997264

[24] Ozdemir BC Sflomos G Brisken C . The challenges of modeling hormone receptor-positive breast cancer in mice. Endocr Relat Cancer. (2018) 25:R319–30. doi: 10.1530/ERC-18-0063 29563191

[25] Buqué A Bloy N Perez-Lanzón M Iribarren K Humeau J Pol JG . Immunoprophylactic and immunotherapeutic control of hormone receptor-positive breast cancer. Nat Commun. (2020) 11:3819. doi: 10.1038/s41467-020-17644-0 32732875 PMC7393498

[26] Perez-Lanzon M Carbonnier V Cordier P De Palma FDE Petrazzuolo A Klein C . New hormone receptor-positive breast cancer mouse cell line mimicking the immune microenvironment of anti-PD-1 resistant mammary carcinoma. J Immunother Cancer. (2023) 11. doi: 10.1136/jitc-2023-007117 37344100 PMC10314679

[27] Kasikara C Davra V Calianese D Geng K Spires TE Quigley M . Pan-TAM tyrosine kinase inhibitor BMS-777607 enhances anti-PD-1 mAb efficacy in a murine model of triple-negative breast cancer. Cancer Res. (2019) 79:2669–83. doi: 10.1158/0008-5472.can-18-2614 30877108

[28] Ciavattone NG Guan N Farfel A Stauff J Desmond T Viglianti BL . Evaluating immunotherapeutic outcomes in triple-negative breast cancer with a cholesterol radiotracer in mice. JCI Insight. (2024) 9. doi: 10.1172/jci.insight.175320 38502228 PMC11141879

[29] Andrade FO Jin L Clarke R Wood I Dutton M Anjorin C . Social isolation activates dormant mammary tumors, and modifies inflammatory and mitochondrial metabolic pathways in the rat mammary gland. Cells. (2023) 12. doi: 10.3390/cells12060961 36980301 PMC10047513

[30] Clarke KR . Non-parametric multivariate analyses of changes in community structure. Aust J Ecol. (1993) 18:117–43. doi: 10.1111/j.1442-9993.1993.tb00438.x 40046247

[31] Anderson MJ Walsh DCI . PERMANOVA, ANOSIM, and the Mantel test in the face of heterogeneous dispersions: what null hypothesis are you testing? Ecol Monogr. (2013) 83:557–74. doi: 10.1890/12-2010.1

[32] Warton DI Wright ST Wang Y . Distance-based multivariate analyses confound location and dispersion effects. Methods Ecol Evol. (2012) 3:89–101. doi: 10.1111/j.2041-210x.2011.00127.x 40046247

[33] Chen IX Newcomer K Pauken KE Juneja VR Naxerova K Wu MW . A bilateral tumor model identifies transcriptional programs associated with patient response to immune checkpoint blockade. Proc Natl Acad Sci. (2020) 117:23684–93. doi: 10.1073/pnas.2002806117 32907939 PMC7519254

[34] Chang EC Charn TH Park SH Helferich WG Komm B Katzenellenbogen JA . Estrogen receptors alpha and beta as determinants of gene expression: influence of ligand, dose, and chromatin binding. Mol Endocrinol. (2008) 22:1032–43. doi: 10.1210/me.2007-0356 18258689 PMC2366177

[35] Barkhem T Carlsson B Nilsson Y Enmark E Gustafsson J-Å Nilsson S . Differential response of estrogen receptor α and estrogen receptor β to partial estrogen agonists/antagonists. Mol Pharmacol. (1998) 54:105–12. doi: 10.1124/mol.54.1.105 9658195

[36] Kuiper GG Lemmen JG Carlsson B Corton JC Safe SH van der Saag PT . Interaction of estrogenic chemicals and phytoestrogens with estrogen receptor beta. Endocrinology. (1998) 139:4252–63. doi: 10.1210/endo.139.10.6216 9751507

[37] Oner G Broeckx G Van Berckelaer C Zwaenepoel K Altintas S Canturk Z . The immune microenvironment characterisation and dynamics in hormone receptor-positive breast cancer before and after neoadjuvant endocrine therapy. Cancer Med. (2023) 12:17901–13. doi: 10.1002/cam4.6425 37553911 PMC10524081

[38] Hulskotter KJ Allnoch L Hansmann F Schmidtke D Rohn K Flugel A . Double-edged effects of tamoxifen-in-oil-gavage on an infectious murine model for multiple sclerosis. Brain Pathol. (2021) 31:e12994. doi: 10.1111/bpa.12994 34137105 PMC8549030

[39] Larsson AM Roxå A Leandersson K Bergenfelz C . Impact of systemic therapy on circulating leukocyte populations in patients with metastatic breast cancer. Sci Rep. (2019) 9:13451. doi: 10.1038/s41598-019-49943-y 31530882 PMC6748932

[40] Huang B Warner M Gustafsson J . Estrogen receptors in breast carcinogenesis and endocrine therapy. Mol Cell Endocrinol. (2015) 418:240–4. doi: 10.1016/j.mce.2014.11.015 25433206

[41] Sivan A Corrales L Hubert N Williams JB Aquino-Michaels K Earley ZM . Commensal Bifidobacterium promotes antitumor immunity and facilitates anti-PD-L1 efficacy. Science. (2015) 350:1084–91. doi: 10.1126/science.aac4255 PMC4873287 26541606

[42] McCulloch JA Davar D Rodrigues RR Badger JH Fang JR Cole AM . Intestinal microbiota signatures of clinical response and immune-related adverse events in melanoma patients treated with anti-PD-1. Nat Med. (2022) 28:545–56. doi: 10.1038/s41591-022-01698-2 35228752 PMC10246505

[43] Wastyk HC Fragiadakis GK Perelman D Dahan D Merrill BD Yu FB . Gut-microbiota-targeted diets modulate human immune status. Cell. (2021) 184:4137–53. doi: 10.1016/j.cell.2021.06.019 34256014 PMC9020749

[44] Tuck CJ De Palma G Takami K Brant B Caminero A Reed DE . Nutritional profile of rodent diets impacts experimental reproducibility in microbiome preclinical research. Sci Rep. (2020) 10:17784. doi: 10.1038/s41598-020-74460-8 33082369 PMC7575541

[45] Schipper L Tims S Timmer E Lohr J Rakhshandehroo M Harvey L . Grain versus AIN: common rodent diets differentially affect health outcomes in adult C57BL/6j mice. PloS One. (2024) 19:e0293487. doi: 10.1371/journal.pone.0293487 38512932 PMC10956799

[46] Chalova P Tazky A Skultety L Minichova L Chovanec M Ciernikova S . Determination of short-chain fatty acids as putative biomarkers of cancer diseases by modern analytical strategies and tools: a review. Front Oncol. (2023) 13:1110235. doi: 10.3389/fonc.2023.1110235 37441422 PMC10334191

[47] Zhang M Wei Z Wei B Lai C Zong G Tao E . Microbiota-derived urocanic acid triggered by tyrosine kinase inhibitors potentiates cancer immunotherapy efficacy. Cell Host Microbe. (2025) 33:915–931.e9. doi: 10.1016/j.chom.2025.04.022 40441145

[48] De Oliveira Andrade FS Jin L Ozgul-Onal M McDermott M Kenanoglu S Andrade de Oliveira K . Gut microbiome modulates breast cancer risk in socially isolated mice. Breast Cancer Res. (2026). doi: 10.1186/s13058-026-02292-x PMC13312772 42069657

[49] Le Naour A Rossary A Vasson MP . EO771, is it a well-characterized cell line for mouse mammary cancer model? Limit and uncertainty. Cancer Med. (2020) 9:8074–85. doi: 10.1002/cam4.3295 33026171 PMC7643677

[50] Yuan B Clark CA Wu B Yang J Drerup JM Li T . Estrogen receptor beta signaling in CD8(+) T cells boosts T cell receptor activation and antitumor immunity through a phosphotyrosine switch. J Immunother Cancer. (2021) 9. doi: 10.1136/jitc-2020-001932 33462142 PMC7816924

[51] Zhu B Tse LA Wang D Koka H Zhang T Abubakar M . Immune gene expression profiling reveals heterogeneity in luminal breast tumors. Breast Cancer Res. (2019) 21:147. doi: 10.1186/s13058-019-1218-9 31856876 PMC6924001

[52] Terranova-Barberio M Pawlowska N Dhawan M Moasser M Chien AJ Melisko ME . Exhausted T cell signature predicts immunotherapy response in ER-positive breast cancer. Nat Commun. (2020) 11:3584. doi: 10.1038/s41467-020-17414-y 32681091 PMC7367885

[53] Tietscher S Wagner J Anzeneder T Langwieder C Rees M Sobottka B . A comprehensive single-cell map of T cell exhaustion-associated immune environments in human breast cancer. Nat Commun. (2023) 14:98. doi: 10.1038/s41467-022-35238-w 36609566 PMC9822999

[54] Rugo HS Delord JP Im SA Ott PA Piha-Paul SA Bedard PL . Safety and antitumor activity of pembrolizumab in patients with estrogen receptor-positive/human epidermal growth factor receptor 2-negative advanced breast cancer. Clin Cancer Res. (2018) 24:2804–11. doi: 10.1158/1078-0432.ccr-17-3452 29559561

[55] Harris HR Bergkvist L Wolk A . An estrogen-associated dietary pattern and breast cancer risk in the Swedish Mammography Cohort. Int J Cancer. (2015) 137:2149–54. doi: 10.1002/ijc.29586 25924604

[56] Guinter MA McLain AC Merchant AT Sandler DP Steck SE . A dietary pattern based on estrogen metabolism is associated with breast cancer risk in a prospective cohort of postmenopausal women. Int J Cancer. (2018) 143:580–90. doi: 10.1002/ijc.31387 29574860 PMC6019153

[57] Zhao Y Liu Z Qin L Wang T Bai O . Insights into the mechanisms of Th17 differentiation and the Yin-Yang of Th17 cells in human diseases. Mol Immunol. (2021) 134:109–17. doi: 10.1016/j.molimm.2021.03.010 33756352

[58] Stockinger B Omenetti S . The dichotomous nature of T helper 17 cells. Nat Rev Immunol. (2017) 17:535–44. doi: 10.1038/nri.2017.50 28555673

[59] Kalim UU Biradar R Junttila S Khan MM Tripathi S Khan MH . A proximal enhancer regulates RORA expression during early human Th17 cell differentiation. Clin Immunol. (2024) 264:110261. doi: 10.1016/j.clim.2024.110261 38788884

[60] Xiong G Wang C Evers BM Zhou BP Xu R . RORα suppresses breast tumor invasion by inducing SEMA3F expression. Cancer Res. (2012) 72:1728–39. doi: 10.1158/0008-5472.can-11-2762 22350413 PMC3319846

[61] Oh SK Kim D Kim K Boo K Yu YS Kim IS . RORα is crucial for attenuated inflammatory response to maintain intestinal homeostasis. Proc Natl Acad Sci USA. (2019) 116:21140–9. doi: 10.1073/pnas.1907595116 31570593 PMC6800319

[62] He S Yu J Sun W Sun Y Tang M Meng B . A comprehensive pancancer analysis reveals the potential value of RAR-related orphan receptor C (RORC) for cancer immunotherapy. Front Genet. (2022) 13:969476. doi: 10.3389/fgene.2022.969476 36186454 PMC9520743

[63] Xia L Tian E Yu M Liu C Shen L Huang Y . RORγt agonist enhances anti-PD-1 therapy by promoting monocyte-derived dendritic cells through CXCL10 in cancers. J Exp Clin Cancer Res. (2022) 41:155. doi: 10.1186/s13046-022-02289-2 35459193 PMC9034499

[64] Lalle G Lautraite R Bouherrou K Plaschka M Pignata A Voisin A . NF-κB subunits RelA and c-Rel selectively control CD4+ T cell function in multiple sclerosis and cancer. J Exp Med. (2024) 221. doi: 10.1084/jem.20231348 38563819 PMC10986815

[65] Schraml BU Hildner K Ise W Lee WL Smith WA Solomon B . The AP-1 transcription factor Batf controls T(H)17 differentiation. Nature. (2009) 460:405–9. doi: 10.1038/nature08114 19578362 PMC2716014

[66] Seo H González-Avalos E Zhang W Ramchandani P Yang C Lio CJ . BATF and IRF4 cooperate to counter exhaustion in tumor-infiltrating CAR T cells. Nat Immunol. (2021) 22:983–95. doi: 10.1038/s41590-021-00964-8 34282330 PMC8319109

[67] Sonnenblick A Im SA Lee KS Tan A Telli M Strulov Shachar S . 267P Phase Ib/II open-label, randomized evaluation of second- or third-line (2L/3L) atezolizumab (atezo) + entinostat (entino) in MORPHEUS-HR+ breast cancer (M-HR+BC). Ann Oncol. (2021) 32:S479. doi: 10.1016/j.annonc.2021.08.550 38826717

[68] Alaluf E Shalamov MM Sonnenblick A . Update on current and new potential immunotherapies in breast cancer, from bench to bedside. Front Immunol. (2024) 15:1287824. doi: 10.3389/fimmu.2024.1287824 38433837 PMC10905744

