# Supplementary material for: Isoflavones impair anti-PD1 efficacy in breast cancer, regardless of dietary fiber or fecal short-chain fatty acid levels
Source: Front Immunol. 2026 Jul 6;17:1835466. doi: 10.3389/fimmu.2026.1835466 (PMC13381617; doi:10.3389/fimmu.2026.1835466)
Supplement: Supplementary file 1 [file DataSheet1.docx]

**
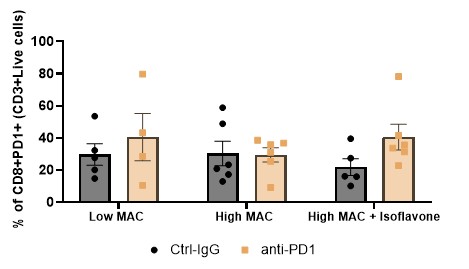
**

**Supplementary Figure 1.** **Effect of anti-PD1 on CD8+PD1+ T cells in mice fed different diets.** Frequency of CD8+PD1+ cells, in tumors of mice fed low-MAC, high-MAC or high-MAC isoflavone (MACi) diets and treated with anti-PD1 or Ctrl-IgG. Cells were gated on live cells, followed by CD3+ cells, CD8+ cells and PD1+. Gates were determined based on FMO staining. There were 4-6 tumors in each group. Data are shown as mean ± SEM.

**Supplementary table 1.** Composition of low-MAC, high-MAC and high MACi diets fed to mice.

|  | **Diet (AIN93G)** | **LabDiet 5V5M** | **LabDiet 5058D** |
| --- | --- | --- | --- |
| Primary ingredient | Corn sugar | Ground Wheat | Ground Wheat |
| Secondary ingredient | Casein | Ground Corn, Corn Gluten Meal | Ground Corn, Dehulled Soybean Meal |
| % min. protein | 17.9 | 18 | 20 |
| % min. crude fat | 7 | 8 | 9 |
| % max. cellulose | 4.8 | 5 | 4.0 |
| % kcal from fat | 17.2 | 21.5 | 21.5 |
| Kcal/gm | 3.8 | 3.8 | 3.8 |
| **Isoflavone content** | **<5 ppm** | **50 ppm** | **125-275 ppm*** |

* Ranges of isoflavones reported in rodent LabDiet 5058D. (1, 2).

** **Qualitative Fiber Profile Note:** The low-MAC diet utilizes a purified formula where cellulose serves as the sole, highly insoluble, and poorly fermentable fiber source. In contrast, the high-MAC and high-MACi diets are grain- and legume-based matrices. Ground wheat provides a multi-functional carbohydrate matrix. This includes highly fermentable substrates, spanning both **s**oluble non-starch polysaccharides and oligosaccharides (predominantly arabinoxylans and fructans, alongside lower native fractions of β-glucans) and insoluble resistant starches. These components escape upper gastrointestinal digestion to serve as primary prebiotic substrates for SCFA-producing gut microbiota (3-5). Concurrently, ground wheat and ground corn introduce complex insoluble structural components, primarily cellulose and hemicellulose, along with minor fractions of lignin, which collectively resist rapid microbial fermentation, influence intestinal transit time, and modulate matrix accessibility (3-6). Additionally, the dehulled soybean meal in the high-MACi diet provides supplemental soluble pectins and oligosaccharides (e.g., raffinose and stachyose), offering a highly diverse substrate profile to support robust microbial fermentation and SCFA generation (3,4,7,8,9)

**References:**

(1) Thigpen JE, Setchell KD, Ahlmark KB, Locklear J, Spahr T, Caviness GF, et al. Phytoestrogen content of purified, open- and closed-formula laboratory animal diets. Lab Anim Sci 1999;49:530-536.

(2) Brown NM, Setchell KD. Animal models impacted by phytoestrogens in commercial chow: implications for pathways influenced by hormones. Lab Invest 2001;81:735-747.

(3) Knudsen, K. E. B. (1997). Carbohydrate and lignin contents of plant materials used in animal feeding. *Animal Feed Science and Technology*, 67(4), 319-338.

(4) Shewry, P. R., & Hey, S. J. (2015). The contribution of wheat to human diet and health. *Food and Energy Security*, 4(3), 178-202.

(5) Williams, B.A., Mikkelsen, D., Flanagan, B.M., Gidley, M.J., Dietary fibre”: moving beyond the “soluble/insoluble” classification for monogastric nutrition, with an emphasis on humans and pigs. J Anim Sci Biotechnol. 2019 May 24;10:45. doi: [10.1186/s40104-019-0350-9](https://doi.org/10.1186/s40104-019-0350-9)

(6) Modasia, A.A., Spiller, R.C., Warren F.J. Role of fermentation-resistant and non-fermentable fibers in gastrointestinal health: mechanisms, benefits, and challenges.
Crit Rev Food Sci Nutr 2026 Jan 21:1-17. doi: 10.1080/10408398.2025.2612556.

(7) Pascale, N., Gu, F., Larsen, N., Jepersen, L., Respondek, F. The Potential of Pectins to Modulate the Human Gut Microbiota Evaluated by In Vitro Fermentation: A Systematic Review. Nutrients. 2022 Sep 2;14(17):3629. doi: [10.3390/nu14173629](https://doi.org/10.3390/nu14173629)

(8) Elango, D., et al. Raffinose Family Oligosaccharides: Friend or Foe for Human and Plant Health? Front Plant Sci. 2022 Feb 17;13:829118. doi: [10.3389/fpls.2022.829118](https://doi.org/10.3389/fpls.2022.829118). PMID: [35251100](https://pubmed.ncbi.nlm.nih.gov/35251100/).

(9) Sanyal, R., et al. Optimizing raffinose family oligosaccharides content in plants: A tightrope walk. Front Plant Sci 2023 Mar 28:14:1134754. doi: 10.3389/fpls.2023.1134754. eCollection 2023.

**Supplementary table 2.** Primers used in quantitative real-time PCR.

| Gene | Sequence |
| --- | --- |
| *Batf_*Forward | 5’-CCCCCTAGCAGTCAAGAAGG-3’ |
| *Batf_*Reverse | 5’-CATCAGATGAGTCCTGTTTGCCA-3’ |
| *Jun_* Forward | 5’-CCTTCTACGACGATGCCCTC -3’ |
| *Jun_* Reverse | 5’-AGAAGGTCCGAGTTCTTGGC -3’ |
| *Rela_* Forward | 5’- CCAGACACAGATGATCGCCA-3’ |
| *Rela_* Reverse | 5’- TTTCGGGTAGGCACAGCAAT-3’ |
| *Rora_* Forward | 5’- TGGTGTCATTACGTGTGAAGG-3’ |
| *Rora_* Reverse | 5’- CATTTCTGCAGCCGACAATG-3’ |
| *Rorc_* Forward | 5’- TTTGAAGGCAAATACGGTGGTG -3’ |
| *Rorc_* Reverse | 5’- CCGTGTAGAGGGCAATCTCATC -3’ |
| *Smad4_* Forward | 5’-TTGTCTCACCTGGAATTGATCTC -3’ |
| *Smad4_* Reverse | 5’-GGTGTTGGATGGTTTGAATCG -3’ |
| *Tbp_*Forward | 5’-AGGATGCTCTAGGGAAGATCTGAG -3’ |
| *Tbp_*Reverse | 5’-GAGCATAAGGTGGAAGGCTGTT -3’ |

**Supplementary table 3**. The list of 123 genes that differentially expressed in mice fed high MAC isoflavone diet and treated with TAM+anti-PD1 versus those treated with Ctrl-IgG, anti-PD1 or TAM.

| **gene_symbol** | **FDR** | **FC** |  | **gene_symbol** | **FDR** | **FC** |
| --- | --- | --- | --- | --- | --- | --- |
| Akt3 | 0.03134031 | -2.9942669 |  | Tnfrsf12A | 0.02124425 | -3.0036473 |
| Cdkn1A | 0.0199046 | -2.1101596 |  | Plaur | 0.0270946 | -2.0717859 |
| Tlr6 | 0.01073647 | 2.05462763 |  | Tollip | 0.04972993 | -1.4523215 |
| Nod1 | 0.03903679 | 1.55727528 |  | Axl | 0.04481423 | -1.733882 |
| Batf | 0.01195471 | -1.7089382 |  | Pdgfc | 0.00409181 | 2.32891715 |
| Txnip | 0.01955193 | 1.69600779 |  | Map2K1 | 0.0437883 | -1.6143939 |
| Cxcr6 | 0.0342522 | 1.98533699 |  | Map2K2 | 0.04228564 | -1.5785764 |
| Masp2 | 0.0316699 | 1.97361672 |  | Psmd7 | 0.0316699 | -1.7981717 |
| Ccr9 | 0.03695333 | 1.79094519 |  | Bax | 0.0029906 | -2.1366302 |
| Il24 | 0.00113831 | -3.6425565 |  | Rag1 | 0.04756742 | 1.97157604 |
| Chuk | 0.0270946 | -1.6819717 |  | Il22Ra1 | 0.01733093 | 1.99186041 |
| Gbp5 | 0.02719992 | -1.7846203 |  | Rela | 0.02244261 | -2.057423 |
| Il22Ra2 | 0.01059193 | -2.0772873 |  | Bcl2L1 | 0.03975519 | -1.700731 |
| Cd200R1 | 0.03903679 | 2.02582592 |  | Tnfrsf17 | 0.0346573 | 2.14418947 |
| Csf2Rb | 0.0270946 | 1.90563654 |  | Rora | 0.0316699 | 1.89365901 |
| Il34 | 0.01955193 | -2.2653999 |  | Rorc | 0.02930857 | 2.87728025 |
| Cybb | 0.02539186 | -1.8217281 |  | Ccl1 | 0.02741334 | 1.87824009 |
| Cyld | 0.01733093 | -1.945049 |  | Ccl5 | 0.04935725 | -2.6515966 |
| Cd55 | 0.01741418 | 2.91268299 |  | Ccl19 | 0.0346573 | 3.29419715 |
| Dusp6 | 0.0225174 | -1.8201548 |  | Cxcl12 | 0.01299462 | 4.07911624 |
| Egfr | 0.01320504 | 1.88202304 |  | Card9 | 0.01059193 | 2.3948437 |
| Egr3 | 0.02772172 | -3.3276288 |  | Il25 | 0.04573135 | 1.66543076 |
| Elane | 0.0181291 | 1.93334906 |  | Stat3 | 0.02821217 | -1.632427 |
| Ep300 | 0.03957984 | -1.7079085 |  | Stat5B | 0.04052037 | -1.800092 |
| Ets1 | 0.01431796 | -2.5627486 |  | Tek | 0.01580827 | 2.09894845 |
| Ewsr1 | 0.0316699 | -1.9086992 |  | Tfrc | 0.01320504 | -5.0713677 |
| Fn1 | 0.0316699 | 3.09280278 |  | Tgfbr2 | 0.04756742 | 1.86792552 |
| Ddx58 | 0.0346573 | -1.4784916 |  | Thbd | 0.00100765 | 3.73852104 |
| Fyn | 0.0346573 | -1.5581132 |  | Tlr2 | 0.0342522 | 2.06363819 |
| Cyfip2 | 0.02131851 | -2.6619783 |  | Serping1 | 0.01059193 | 3.71862552 |
| Il17B | 0.0095343 | 2.46954978 |  | Tlr5 | 0.00132925 | 2.54548315 |
| Ifnl2 | 0.03975519 | 2.04001703 |  | C3 | 0.0346573 | 3.23884991 |
| Cxcr3 | 0.04477481 | 1.83240324 |  | Traf2 | 0.04756742 | -1.6333039 |
| Angpt1 | 0.00554772 | 2.4637578 |  | C4B | 0.03003791 | 3.03930047 |
| Icos | 0.04631885 | 1.52072327 |  | C6 | 0.01404783 | 3.68269664 |
| Tbx21 | 0.04223251 | 1.64476578 |  | Twist1 | 0.01299462 | -2.5543036 |
| Cfh | 0.00773692 | 4.1294774 |  | Tnfrsf4 | 0.0316699 | 2.20573655 |
| Birc5 | 0.02244261 | -2.515878 |  | C8B | 0.01404783 | 2.12252934 |
| Ifit1 | 0.0095343 | -2.6394151 |  | Vcam1 | 0.01059193 | -2.3366487 |
| Ifna2 | 0.04934204 | 2.58848463 |  | Vegfc | 0.02090285 | -1.7464871 |
| Ticam2 | 0.01712508 | 2.21858155 |  | Zap70 | 0.02816112 | 1.58444052 |
| Il1Rap | 0.0199046 | -3.4620155 |  | Il1R2 | 0.02131851 | 2.29312214 |
| Il2Rg | 0.02131851 | -2.5050756 |  | Casp3 | 0.00542368 | -2.3941561 |
| Il3 | 0.02090285 | -2.7045925 |  | Runx1 | 0.0346573 | -1.7303273 |
| Il5Ra | 0.02600494 | 1.72925413 |  | Runx3 | 0.0181291 | 1.96185087 |
| Il13 | 0.0316699 | 1.88307711 |  | Socs1 | 0.01299462 | -2.6978036 |
| Tnfrsf9 | 0.02106348 | -2.0642587 |  | Tnfsf13 | 0.03003791 | 2.09252296 |
| Irak1 | 0.03903679 | -1.5703342 |  | Fadd | 0.04507881 | 1.69166466 |
| Itga6 | 0.02131851 | -2.7912631 |  | Tnfrsf11A | 0.0346573 | 1.81573747 |
| Irf4 | 0.02090285 | 2.51435181 |  | Il1Rl2 | 0.02131851 | 2.25295503 |
| Itgb1 | 0.02090285 | -2.0628777 |  | Il18R1 | 0.04152203 | 1.76215977 |
| Jak1 | 0.04573135 | -1.4701512 |  | Nrp1 | 0.02090285 | 2.03950427 |
| Jun | 0.03957984 | -1.9056501 |  | Bcl10 | 0.03134031 | -1.6199738 |
| Lbp | 0.02516831 | 4.08015137 |  | Ccnd3 | 0.04507881 | 1.44101994 |
| Smad4 | 0.02304796 | -1.7285089 |  | Cd3D | 0.04507881 | 1.52049797 |
| Maf | 0.0316699 | 2.03830708 |  | Il1Rl1 | 0.01933978 | 1.74257391 |
| Mmp9 | 0.0199046 | 2.43676909 |  | Cd7 | 0.0076462 | 2.28855847 |
| Myc | 0.00773693 | -3.9492388 |  | Cd14 | 0.03003791 | 1.68638804 |
| Nfatc4 | 0.0076462 | 1.97733845 |  | Cd19 | 0.04972993 | 2.15707361 |
| Nfkb2 | 0.02244261 | -2.2146847 |  | Cd33 | 0.02131851 | 1.93077452 |
| Il22 | 0.00100765 | -3.3555131 |  | Ikbke | 0.04087209 | 2.09353187 |
| Foxp3 | 0.03975519 | -2.0318902 |  |  |  |  |
